# Determinants of Vaccination and Willingness to Vaccinate against COVID-19 among Pregnant and Postpartum Women during the Third Wave of the Pandemic: A European Multinational Cross-Sectional Survey

**DOI:** 10.3390/v15051090

**Published:** 2023-04-29

**Authors:** Emeline Maisonneuve, Eva Gerbier, Fatima Tauqeer, Léo Pomar, Guillaume Favre, Ursula Winterfeld, Anneke Passier, Alison Oliver, David Baud, Hedvig Nordeng, Michael Ceulemans, Alice Panchaud

**Affiliations:** 1 Institute of Primary Health Care (BIHAM), University of Bern, 3012 Bern, Switzerland; 2 Materno-Fetal and Obstetrics Research Unit, Department “Femme-Mère-Enfant”, Lausanne University Hospital and University of Lausanne, 1011 Lausanne, Switzerland; leo.pomar@chuv.ch (L.P.); guillaume.favre@chuv.ch (G.F.); david.baud@chuv.ch (D.B.); 3 Service of Pharmacy, Lausanne University Hospital and University of Lausanne, 1011 Lausanne, Switzerland; eva.gerbier@chuv.ch; 4 Pharmacoepidemiology and Drug Safety Research Group, Department of Pharmacy, PharmaTox Strategic Research Initiative, Faculty of Mathematics and Natural Sciences, University of Oslo, 0316 Oslo, Norway; fatima.tauqeer@farmasi.uio.no (F.T.); h.m.e.nordeng@farmasi.uio.no (H.N.); 5 School of Health Sciences (HESAV), University of Applied Sciences and Arts Western Switzerland, 1011 Lausanne, Switzerland; 6 Swiss Teratogen Information Service, Clinical pharmacology Service, Lausanne University Hospital and University of Lausanne, 1011 Lausanne, Switzerland; ursula.winterfeld@chuv.ch; 7 Teratology Information Service, Pharmacovigilance Centre Lareb, 5237 MH ’s Hertogenbosch, The Netherlands; a.passier@lareb.nl (A.P.); michael.ceulemans@kuleuven.be (M.C.); 8 UK Teratology Information Service, Newcastle upon Tyne Hospitals, NHS Foundation Trust and the UK Health Security Agency, Newcastle upon Tynes NE2 4AB, UK; alison.oliver12@nhs.net; 9 Department of Pharmaceutical and Pharmacological Sciences, KU Leuven, 3000 Leuven, Belgium; 10 L-C&Y, KU Leuven Child & Youth Institute, 3000 Leuven, Belgium

**Keywords:** vaccination hesitancy, vaccination willingness, COVID-19 vaccines, COVID-19, SARS-CoV-2, pregnancy, postpartum, breastfeeding

## Abstract

With COVID-19 vaccination hesitancy at around 50% in the obstetric population, it is critical to identify which women should be addressed and how. Our study aimed to assess COVID-19 vaccination willingness among pregnant and postpartum women in Europe and to investigate associated determinants. This study was a cross-sectional, web-based survey conducted in Belgium, Norway, Switzerland, The Netherlands, and United Kingdom (UK) in June–August 2021. Among 3194 pregnant women, the proportions of women vaccinated or willing to be vaccinated ranged from 80.5% in Belgium to 21.5% in Norway. The associated characteristics were country of residence, chronic illness, history of flu vaccine, trimester of pregnancy, belief that COVID-19 is more severe during pregnancy, and belief that the COVID-19 vaccine is effective and safe during pregnancy. Among 1659 postpartum women, the proportions of women vaccinated or willing to be vaccinated ranged from 86.0% in the UK to 58.6% in Switzerland. The associated determinants were country of residence, chronic illness, history of flu vaccine, breastfeeding, and belief that the COVID-19 vaccine is safe during breastfeeding. Vaccine hesitancy in the obstetric population depends on medical history and especially on the opinion that the vaccine is safe and on the country of residence.

## 1. Introduction

Vaccination against coronavirus disease 2019 (COVID-19) is of particular importance to pregnant and lactating women. COVID-19 is associated with a higher risk of severe illness in pregnant women than in similarly aged non-pregnant women and those with a higher risk of adverse pregnancy outcomes when compared to uninfected pregnant women [1,2,3]. A meta-analysis, based on studies published between December 2020 and January 2022, has shown that the effectiveness of the mRNA vaccination against SARS-CoV-2 infection seven days after the second dose was 89.5% in pregnant women (95% confidence interval (CI) 69.0–96.4%; 18,828 vaccinated pregnant women; I^2^ = 73.9%). This meta-analysis also found that the risk of stillbirth was significantly lower in the vaccinated cohort by 15%. Moreover, there was no evidence of an increased risk of adverse maternal, pregnancy or neonatal outcomes after the prenatal COVID-19 vaccination, supporting the safety of COVID-19 vaccines in pregnancy [4]. Vaccinating pregnant women against COVID-19 is also beneficial for their unborn infants, with a reduced risk of hospitalization for COVID-19 before 6 months of age, including for critical illness [5]. Regarding vaccination during breastfeeding, the breast milk of vaccinated individuals has shown to contain SARS-CoV-2-specific antibodies and T cells, which may benefit the breastfed infant’s developing immune system [6,7].

Despite growing evidence of the benefits and safety of vaccination in pregnant and breastfeeding women, vaccination hesitancy in the obstetric population is highly prevalent and represented 48.4% in 2020–2022 [8]. The determinants positively associated with vaccination willingness found in meta-analyses, encompassing an obstetric population, were age >35, white ethnic origin, higher school education, previous influenza/dtaP vaccination, employment, third trimester of pregnancy, and sufficient information about the SARS-CoV-2 vaccine [8,9,10,11,12]. As vaccination in pregnancy continues to be important, there is a need to improve the willingness of pregnant and breastfeeding women to vaccinate against COVID-19 in order to protect themselves and their newborns [5]. Therefore, it is critical to identify which women should be specifically targeted with vaccination campaigns or during counselling in healthcare practices [13].

Thus, our study aimed (1) to assess COVID-19 vaccination status and willingness among pregnant and postpartum women in five European countries during the third wave of the pandemic; (2) to investigate the socio-demographic, medical history, and personal beliefs associated with willingness to vaccinate; and (3) to explore the sources of information that are considered reliable by pregnant and postpartum women.

## 2. Materials and Methods

### 2.1. Study Design and Population

This study is a cross-sectional, web-based survey conducted in Belgium, Norway, Switzerland, The Netherlands, and the United Kingdom (UK) between June and August 2021, i.e., around the third wave of the COVID-19 pandemic. The survey was part of a multinational COVID-19 research project aimed at providing insights into pregnant and postpartum women’s mental health status, perinatal and birth experiences, vaccine acceptance, and medication use [14,15,16]. This European survey is part of the research activities from ENTIS members, which is a global collaborative network of Teratology Information Services, working together to promote safe medication use in pregnancy. Publications arising from this collaborative network, including studies on the effect of COVID-19 pandemic on pregnancy, are available at the ENTIS website: www.entis-org.eu/publications (accessed on 18 April 2023). The current study focuses on questions related to COVID-19 vaccination status and willingness to be vaccinated.

Pregnant and postpartum women who gave birth in the three months preceding the study period, and who were aged 18 or older, were eligible to participate. Women were classified into two groups according to their obstetrical status, i.e., as pregnant or as postpartum. Participants who did not answer the question on vaccine status were excluded from the study, as well as unvaccinated patients who did not answer the question on vaccine willingness.

### 2.2. Data Collection

An online, anonymous survey was distributed in Belgium, Norway, Switzerland, The Netherlands, and the United Kingdom between 10 June and 22 August 2021. It was divided into two questionnaires, one tailored to pregnant and the other to postpartum women (Supplementary Material S1). Pregnant and postpartum women were recruited via banners on national websites and social networks commonly visited by pregnant women or new mothers (Supplementary Material S2). Women were asked to fill in an anonymous online questionnaire hosted on the KU Leuven Qualtrics survey platform (for the surveys that were distributed in Belgium, Switzerland, The Netherlands, and the UK) and the University of Oslo’s Nettskjema platform (for the Norwegian survey). The survey was based on instruments used in a prior COVID-19 study covering the first wave of the pandemic in 2020 to allow comparison [17,18]. It was slightly modified to accommodate the context and research interests at the time of the study period. The survey was first developed in English and then translated into five additional languages (French, Dutch, Norwegian, German, and Italian). To place the study in the context of the third wave of the pandemic, an overview of national COVID-19 infection and vaccination rates in the general population at the time of the survey and the timeline of successive steps of national recommendations for vaccination of pregnant and breastfeeding women in each country are provided in Supplementary Materials S3 and S4 [19,20,21,22,23,24,25,26].

### 2.3. Primary Outcome

The primary outcome was a composite variable defined as “vaccinated or willing to get vaccinated” which included already vaccinated women and unvaccinated women willing to be vaccinated during pregnancy or breastfeeding. This outcome will be abbreviated to “vaccinated/willing” for ease of reading throughout the manuscript.

Information on the vaccination status and willingness to be vaccinated was collected as follows: pregnant and postpartum women were first asked if they had already been vaccinated against coronavirus (yes/no). Unvaccinated participants were asked whether they were willing to be vaccinated against COVID-19 if they had the opportunity during pregnancy (for pregnant women), or breastfeeding (for postpartum women), with following answer options: “yes”, “no”, and “I do not know”. The women who responded “I do not know” were considered as not willing to be vaccinated.

### 2.4. Covariates

The survey collected information on the participants’ socio-demographic characteristics: i.e., country of residence; maternal age; relationship status; professional status; working in healthcare or not; highest level of education, categorized as low (primary school), medium (high school), and high (beyond high school); medical history (i.e., smoking or chronic disease requiring medication in the last 3 months, including asthma, allergy, hypothyroidism, cardiovascular diseases, diabetes, depression, anxiety, epilepsy, rheumatoid arthritis, inflammatory bowel disease, or any other disease); gestational age, categorized as first trimester (<14^0/7^ weeks), second trimester (14^0/7^–27^6/7^ weeks), and third trimester (≥28^0/7^ weeks); gravidity; and current breastfeeding status in postpartum women. Specific data related to the pandemic were also recorded, selected on the basis of the existing literature: i.e., a history of personal positive COVID-19 testing; suspected or confirmed COVID-19 infection; COVID-19 infection in household or close family; and admission to ICU, if any.

### 2.5. The Studied Determinants

The following potential determinants of COVID-19 vaccination status and or willingness were included based on the existing literature: beliefs regarding the impact of the disease (i.e., belief that COVID-19 is more severe during pregnancy or riskier during breastfeeding); history of flu vaccine received the previous winter; perception of different recommended vaccinations (i.e., effectiveness and safety of conventional vaccines in the general population, during pregnancy and breastfeeding, and the effectiveness and safety of COVID-19 vaccines in the general population or in pregnant and breastfeeding women). The following determinants were added to take into account the COVID-19 context in Spring and Summer of 2021 in the different countries: i.e., sources trusted regarding the information they provided on COVID-19 (public authorities: government and health authorities; healthcare providers, such as general practitioners, obstetricians, midwives, and pharmacists; and non-professional sources, such as family and friends, media, and internet); refusal to be vaccinated against COVID-19 by a family member; opinion on the usefulness of restrictive measures to prevent the pandemic (i.e., using disinfectants, wearing masks, social distance, working from home, curfew, and (semi-)lockdown); and opinion on the usefulness of vaccination to prevent the spread of the pandemic.

### 2.6. Statistical Analyses

First, descriptive statistics were used to analyze women’s socio-demographic, obstetrical, and medical characteristics according to their obstetrical status (i.e., pregnant or postpartum). The proportions of vaccination status, COVID-19 vaccine willingness, and the composite variable were described for each obstetrical status and country.

Univariate analyses and multivariable logistic regressions were performed to identify factors independently related to the primary outcome “vaccinated or willing to get vaccinated”. Socio-demographic characteristics, a history of COVID-19, and medical and obstetrical characteristics, that were found to be associated with vaccination hesitancy in the literature were used in the univariate analyses, alongside beliefs regarding COVID-19, vaccination, and restrictive measures implemented against this virus. Then, only variables that were significant in the univariate analyses were included in the adjusted models. The multicollinearity of the variables was checked using correlation tests: if two covariates were correlated with a coefficient >0.70, one of these variables was removed from the logistic regression. Results were shown as crude odds ratios (OR) and adjusted odds ratios (aOR) and 95% CI. Moreover, beliefs on the impact of COVID-19 infection during pregnancy and breastfeeding were compared according to COVID-19 vaccine willingness. Specific reasons to believe that COVID-19 vaccines are not effective and/or not safe among pregnant and postpartum women were also descriptively presented.

In addition, women’s opinions on the usefulness of restrictive measures and on the safety and effectiveness of conventional and COVID-19 vaccines were displayed and compared graphically using five-level Likert scale plots according to vaccination/willingness and obstetric status. The sources trusted to provide information regarding COVID-19 were also displayed using five-level Likert scale plots according to obstetrical status and country. Finally, the hierarchical clustering of determinants associated with vaccination/willingness in the univariate analyses were depicted in dendrograms using Ward’s method to illustrate the hierarchical relationship between the different determinants in pregnant and postpartum women [27].

The analyses were performed on complete cases. The study findings were reported according to the STROBE guidelines for cross-sectional studies. [28] Statistical analyses were performed using R software version 4.2.0 (22 April 2022). Likert scale plots were created with the R packages “ggplot2” and “likert”.

### 2.7. Sensitivity Analyses

#### 2.7.1. Vaccination Willingness Alone in Pregnant Women

As some women may have been vaccinated before pregnancy but no longer wished to be vaccinated during pregnancy, we first conducted a sensitivity analysis on willingness to be vaccinated during pregnancy only, regardless of vaccination status. Moreover, in Norway only, pregnant women were asked whether they were willing to be vaccinated against COVID-19 during pregnancy if they had the opportunity, regardless of their vaccination status. Chi^2^ tests were performed by row to assess whether there was an association between socio-demographic, medical, and obstetrical characteristics of the participants and the three levels of response regarding their willingness to be vaccinated against COVID-19: “not willing to get vaccinated”, “I do not know”, and “willing to get vaccinated”. Two other advantages of this sensitivity analysis were obtaining a more accurate picture of the levels of vaccine hesitancy and examining the influence of vaccination campaigns starting at different times from one country to another.

#### 2.7.2. Vaccination Status or Willingness to Be Vaccinated in Breastfeeding Women

Since postpartum women who were not breastfeeding may not have the same concerns about vaccine safety for their child as breastfeeding women, we performed a second sensitivity analysis on vaccination or willingness to be vaccinated during breastfeeding among breastfeeding women only. This sensitivity analysis aimed to assess whether breastfeeding status could have an impact on the results observed in the postpartum women group.

### 2.8. Ethical Approval

Electronic informed consent was obtained from participants prior to survey initiation. Ethical approval was waived in Norway, Switzerland, The Netherlands, and the UK as the data were collected anonymously. In Belgium, ethical approval was obtained from the Ethics Committee Research UZ/KU Leuven (S63966; 26 May 2021). All data were stored and handled anonymously.

## 3. Results

### 3.1. Participants

**Figure 1:**
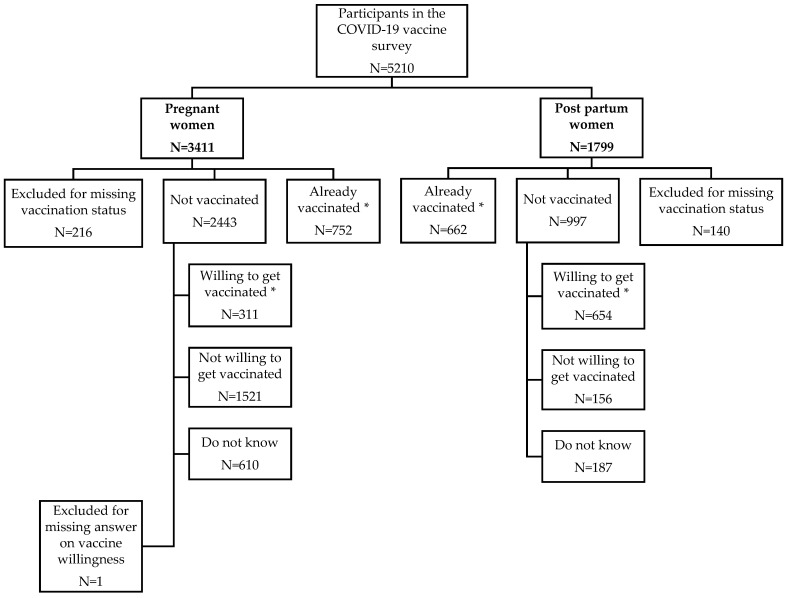
Flowchart of the survey participants according to COVID-19 vaccination status/willingness. * Participants already vaccinated and willing to get vaccinated were merged into the composite variable “Already vaccinated or willing to get vaccinated”.

In total, 5210 women participated in the survey, including 3411 (65.5%) pregnant and 1799 (34.5%) postpartum women. Most responses were collected from Norway (67.0%), followed by Belgium (11.4%), the UK (7.9%), Switzerland (7.4%), and The Netherlands (6.3%) (Table 1). After the exclusion of 216 (6.3%) pregnant and 140 (7.8%) postpartum women who did not answer the questions on COVID-19 vaccination status or willingness, a total of 3194 pregnant women and 1659 postpartum women were included in the analysis on COVID-19 vaccination (Figure 1). In both obstetric populations, 77–78% of the participants had a high educational level. The proportions of pregnant and postpartum women with a chronic illness requiring medication in the previous three months were 36.8% and 26.1%, respectively. The respective proportions of women with a history of a positive COVID-19 test were 4.4 and 5.0%, respectively. More than a quarter of participants were healthcare professionals. Just over half of the pregnant participants were in their third trimester. Among postpartum participants, 91.8% were breastfeeding (Table 1). The characteristics of the 216 pregnant and 140 postpartum women who participated in the survey but were not included in our analysis because they did not answer the question on their vaccination status are presented in Supplementary Table S1. These respondents were more likely to have had COVID-19, to be in the first trimester of pregnancy, and to be less likely to breastfeed than the 3194 pregnant and 1659 postpartum women included in the analysis.

### 3.2. COVID-19 Vaccination Status and Vaccine Willingness

In the cohort of pregnant women (N = 3194), the proportions of women already vaccinated against COVID-19 were 73.8%, 72.5%, 48.0%, 22.4%, and 11.3%, in the UK, Belgium, The Netherlands, Switzerland, and Norway, respectively. The proportions of pregnant women already vaccinated or willing to be vaccinated were 80.5%, 78.5%, 62.6%, 32.1% and 21.5% in Belgium, the UK, The Netherlands, Switzerland, and Norway, respectively. In the cohort of postpartum women (N = 1659), the proportion of participants already vaccinated against COVID-19 were 77.9%, 57.1%, 32.8%, 40.0%, and 50.0% in the UK, Belgium, The Netherlands, Norway, and Switzerland, respectively. The proportions of postpartum women already vaccinated or willing to be vaccinated were 86.0%, 85.7%, 81.0%, 62.6%, and 58.6% in the UK, Belgium, Norway, The Netherlands, and Switzerland, respectively (Table 2).

### 3.3. Determinants Associated with COVID-19 Vaccine Willingness

**Figure 2:**
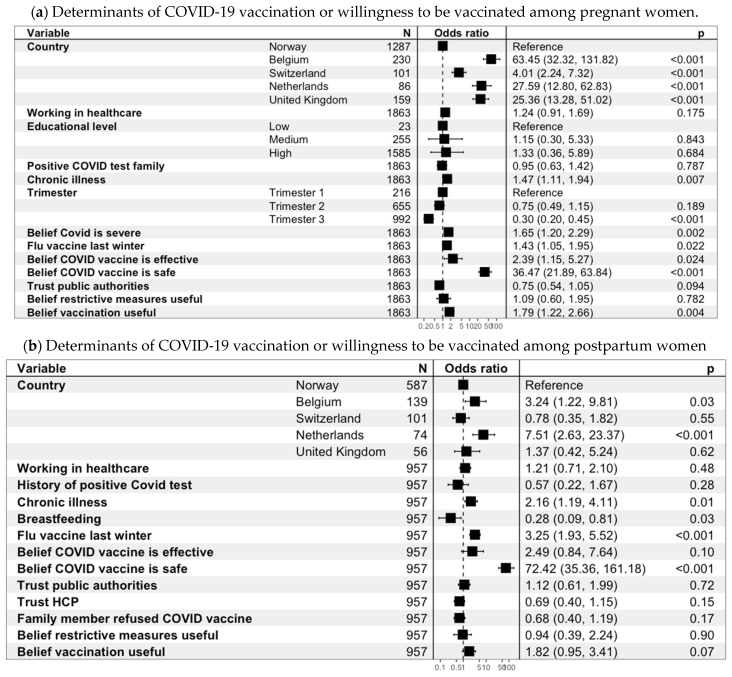
Forest plots of the determinants of COVID-19 vaccination or willingness to be vaccinated among pregnant (**a**) and postpartum women (**b**). These forest plots illustrate the determinants of COVID-19 vaccination or willingness to be vaccinated and the associated adjusted odds ratios. These determinants were included in the multivariable logistic regressions among pregnant and postpartum women (Supplementary Table S2). N: number of observations included in the logistic regressions. HCP: healthcare providers. Among 3194 pregnant women, 1331 observations were deleted due to missingness (2a). Among 1659 postpartum women, 702 observations were deleted due to missingness (2b). When the reference category is not specifically stated, it can be assumed that it is its counterpart.

Among pregnant women, the characteristics associated with COVID-19 vaccination or willingness were country of residence, trimester of pregnancy, chronic illness, history of flu vaccine, belief that COVID-19 is more severe during pregnancy, belief that the COVID-19 vaccine is effective, and belief that the COVID-19 vaccine is safe during pregnancy. More specifically, when compared to women living in Norway, pregnant women living in Belgium (aOR = 63.5, 95%CI 32.3–131.8), The Netherlands (aOR = 27.6, 95%CI 12.8–62.8), the UK (aOR = 25.4, 95%CI 13.3–51.0), and Switzerland (aOR = 4.01, 95%CI 2.24–7.32) were more likely to be already vaccinated or willing to be vaccinated against COVID-19 vaccine. Furthermore, the other characteristics among pregnant women that were positively associated with COVID-19 vaccination/willingness were chronic illness (aOR = 1.47, 95%CI 1.11–1.94), history of flu vaccine (aOR = 1.43, 95%CI 1.05–1.95), and belief that the COVID-19 vaccine is effective (aOR = 2.39, 95%CI 1.15–5.27) and safe during pregnancy (aOR = 36.5, 95%CI 21.9–63.8). The third trimester of pregnancy was negatively associated with COVID-19 vaccination adherence (aOR = 0.30, 95%CI 0.20–0.45) when compared to the first trimester (Figure 2a and Supplementary Table S2).

Among postpartum women, the characteristics associated with COVID-19 vaccination/willingness were country of residence, chronic illness, breastfeeding status, history of flu vaccine, and belief that the COVID-19 vaccine is safe during breastfeeding. More specifically, postpartum women who were living in Belgium (aOR = 3.24, 95%CI 1.22–9.81) and The Netherlands (aOR = 7.51, 95%CI 2.63–23.4) were more vaccinated and/or in favor of receiving a COVID-19 vaccine in the postpartum period compared to women living in Norway. The other characteristics among postpartum women that were positively associated with COVID-19 vaccination/willingness were chronic illness (aOR = 2.16, 95%CI 1.19–4.11), history of flu vaccine (aOR = 3.25, 95%CI 1.93–5.52), and belief that the COVID-19 vaccine is safe during breastfeeding (aOR = 72.4, 95%CI 35.4–161.2). Breastfeeding at the time of survey completion was negatively associated with COVID-19 vaccination/willingness (aOR = 0.28, 95%CI 0.09–0.81) (Figure 2b and Supplementary Table S2).

The associations between the five-level assessments of the safety and effectiveness of the conventional and COVID-19 vaccines in the general population, the pregnant or breastfeeding population, and vaccination status or willingness are visualized using Likert scales in Supplementary Figure S1. The figures show that most women vaccinated/willing to be vaccinated considered the recommended vaccines “very” or “extremely” effective and safe. Likewise, a significant proportion of women not vaccinated/willing to be vaccinated found the same recommended vaccines “not effective” and “not safe” or “not safe at all”; these findings were even more pronounced during the postpartum period than in pregnancy.

The specific reasons why pregnant and postpartum women (breastfeeding or not) considered COVID-19 vaccines to be unsafe are presented in Table 3. Overall, the 12 reasons listed in the questionnaire were consistently chosen more often by pregnant women than breastfeeding and non-breastfeeding women. The two most frequently selected reasons that women considered COVID-19 vaccines to be unsafe were the belief that the long-term effects of these vaccines are not known yet, followed by the belief that some steps of the usual process of vaccine development and approval were not fully completed or bypassed.

### 3.4. Sensitivity Analyses

In the sensitivity analysis on willingness to be vaccinated, regardless vaccination status, the proportions of pregnant women not willing to be vaccinated, those who had not yet decided, and those willing to be vaccinated against COVID-19 were 57.3%, 24.3%, and 18.5%, respectively (Supplementary Table S3). Belgian women were still more willing to be vaccinated than Norwegian women. The proportions of pregnant women who did not know whether they were willing to be vaccinated against COVID-19 were higher in the first trimester and lower in women working in healthcare.

In the second sensitivity analysis that comprised only breastfeeding women instead of all the postpartum women, the factors associated with COVID-19 vaccination/willingness were: living in The Netherlands (aOR = 6.11, 95%CI 2.04–20.1) or in Belgium (aOR = 3.07, 95%CI 1.10–9.98), chronic illness (aOR = 2.14, 95%CI 1.15–4.18), history of flu vaccine (aOR = 3.19, 95%CI 1.86–5.52), and the belief that the COVID-19 vaccine is safe for mother and child while breastfeeding (aOR = 82.0, 95%CI 38.1–194) (Supplementary Table S4). These associations were comparable to those observed in the overall group of postpartum respondents.

### 3.5. Women’s Beliefs about COVID-19 Infection, Measures to Prevent the Pandemic Spread and Their Trust in the Different Sources of Information regarding COVID-19

Pregnant women who had been vaccinated or were willing to be vaccinated were more likely than their counterparts to believe that coronavirus infection could be more severe during pregnancy (86.6% [95%CI 84.4–88.6] versus 70.1% [95% CI 68.1–72.0]). However, among postpartum women, COVID-19 vaccination/willingness was not associated with the belief that breastfeeding may be “risky” to “extremely risky” to the infant if the breastfeeding mother is infected with coronavirus (62.2% [95% CI 59.5–64.8] versus 63.3% [95% CI 57.9–68.4]) (see Supplementary Table S5).

**Figure 3:**
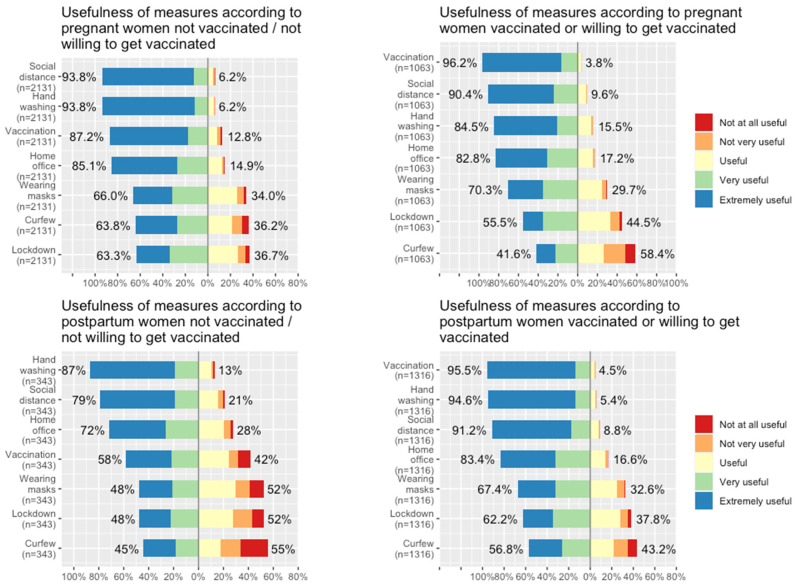
Opinion of pregnant and postpartum women on measures taken to prevent the pandemic spread, by obstetrical status, and COVID-19 vaccination/willingness.

Pregnant and postpartum women who were vaccinated/willing believed that the following measures to prevent the pandemic spread were “very” to “extremely useful”: vaccination (96.2% for pregnant women and 95.5% for postpartum women), disinfection of hands (84.5% and 94.6%), social distancing (90.4% and 91.2%), and working from home (82.8% and 83.4%). In comparison, pregnant and postpartum women not vaccinated/willing believed that the most useful measures to prevent the pandemic spread were disinfection of hands first (93.8% and 87.0%), followed by social distance (93.8% and 79.0%), working from home (85.1% and 72.0%), and then vaccination (87.2% and 58.0%), respectively. Nevertheless, for all groups, these four measures used to prevent the spread of the coronavirus were considered more useful than other measures, such as wearing masks, curfew, and (semi-)lockdown (Figure 3).

**Figure 4:**
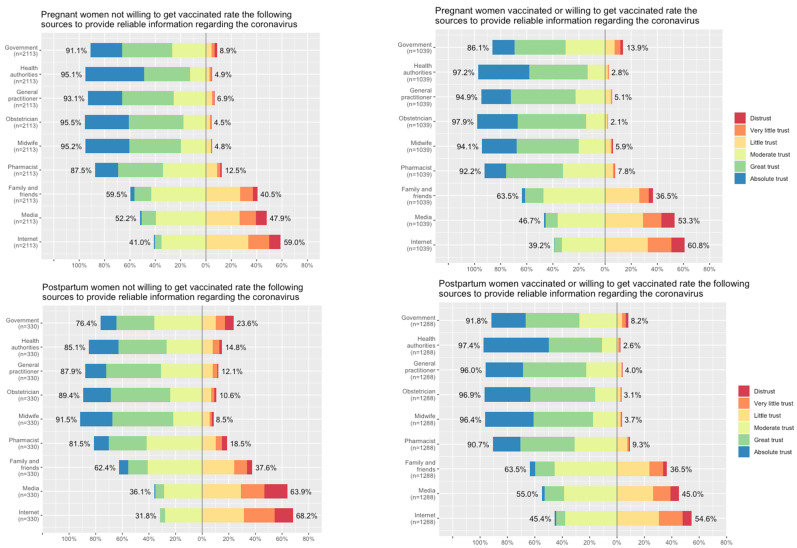
Levels of trust in sources providing information about the coronavirus according to obstetrical status and COVID-19 vaccination/willingness.

Finally, the sources trusted to provide information about the coronavirus were comparable according to vaccination status or willingness and pregnancy or postpartum status (Figure 4). However, differences in trust regarding various sources of information were observed across countries (Supplementary Figure S2). A clustering hierarchical analysis found that the beliefs that the vaccine is effective and safe were in the same cluster for pregnant and postpartum women, revealing that these two variables are more similar to each other than to those in other groups (clusters) (Supplementary Figure S3). These two beliefs were also related to a high educational level among pregnant women. In addition, age above 35 years old, chronic illness, and the belief that COVID-19 is more severe during pregnancy were in the same cluster, which reveals a higher correlation between those characteristics.

## 4. Discussion

### 4.1. Main Findings

In this study, conducted during the third wave of the pandemic in five European countries, the proportions of pregnant women already vaccinated or willing to be vaccinated against COVID-19 ranged between 21.5% in Norway and 80.5% in Belgium. Among postpartum women, the prevalence of vaccination status or willingness ranged from 58.6% in Switzerland to 86.0% in the UK. The following determinants associated with COVID-19 vaccination/willingness among pregnant women were country of residence, trimester of pregnancy, chronic illness, history of flu vaccine, belief that COVID-19 is more severe during pregnancy, belief that the COVID-19 vaccine is effective, and belief that the COVID-19 vaccine is safe during pregnancy. The following positive determinants associated with COVID-19 vaccination/willingness among postpartum women were country of residence, chronic illness, no breastfeeding, history of flu vaccine, and belief that the COVID-19 vaccine is safe during breastfeeding.

Most of our findings regarding the determinants associated with COVID-19 vaccination/willingness are in line with the existing literature, which is more extensive among pregnant than breastfeeding/postpartum women [8,9,10,11,12,29]. In the meta-analysis published by Bhattacharya et al. (eight studies), pregnant women who reported some health comorbidities had a higher prevalence of vaccine acceptance. A Turkish study also found that breastfeeding women at higher risk of severe COVID-19 were more willing to be vaccinated [30]. However, in the meta-analysis published by Bianchi et al., pre-existing chronic disease was not associated with vaccination willingness among pregnant and breastfeeding women [8]. In the general population, being a woman, being under 50 years of age (both criteria related to obstetric population), being single, being unemployed, living in a household with five or more individuals, having an educational attainment lower than an undergraduate degree, having a non-healthcare-related job, and considering COVID-19 vaccines to be unsafe were associated with a higher risk of vaccination hesitancy [31].

Previous influenza/dtaP vaccine uptake or acceptance of the influenza vaccine during pregnancy has also been shown to be associated with COVID-19 vaccine willingness in two meta-analyses and a French multicenter cross-sectional study involving 664 pregnant women (not included in the previous meta-analyses) [8,10,32]. Regarding pregnant women’s willingness to be vaccinated with COVID-19 vaccines, the meta-analysis published by Nindrea et al. found the following determinants: maternal good practice to limit the spread of SARS-CoV-2, favorable perception of the COVID-19 vaccine, and sufficient information about the COVID-19 vaccine [10]. In a Spanish study conducted among 302 pregnant women and 309 healthcare workers investigating factors acting as major decision makers for receiving a vaccination, a recommendation from healthcare workers was the most pivotal influence for pregnant women, with 73% naming it as an influencing factor [33]. An Italian survey revealed that a physician’s recommendation to be vaccinated against COVID-19 is the most important factor in maternal decision making, regardless of geographic, social, or educational context [34]. Similarly, in the previously mentioned French survey performed in Spring 2021, pregnant women who received information from a healthcare provider (HCP) were more likely to accept vaccination [32]. In our study, the most trusted sources among both vaccinated/willing and not vaccinated/not willing were the health authorities and HCPs, but they were even more trusted amongst the vaccinated/willing women. Similarly, in the general population in the UK, the two main determinants associated with vaccination willingness were: older age and a high level of trust in health organizations with crude odds ratios over 20 [35].

Based on our observations, country of residence was a major determinant of COVID-19 vaccination. One potential explanation may be that vaccination/willingness was most likely influenced by the different dates of the initiation of vaccination campaigns in each country as well as the restrictiveness of the inclusion criteria applied at that time (Supplementary Material S3). The recommendation to vaccinate all pregnant women was issued first in Belgium (15 April 2021) and last in Norway (31 January 2022). Belgium issued recommendations to vaccinate all pregnant women regardless of the trimester or the presence of comorbidities. Vaccination recommendations were issued successively on 16 April in the UK at any time during pregnancy, followed by 22 April in The Netherlands, 18 August in Norway, and 14 September 2021 in Switzerland, with a preference for the 2nd and 3rd trimesters [19,20,21,22,23]. The same temporality can be applied to the recommendations for breastfeeding women. Furthermore, in some countries such as Switzerland, at the beginning of the vaccination campaign, vaccination required the signing of a consent form by both an obstetrician/gynecologist and the pregnant woman to certify that the woman had made the decision with her physician, that she had had enough time to think about her decision, and that she had been informed about the (dis)advantages of the vaccine. Our results are consistent with the initiation of the national recommendations to vaccinate pregnant women against COVID-19, highlighting that the late onset and restrictive inclusion criteria for vaccination in health policies might have had a critical impact on vaccination adherence among pregnant women. Belgium was the first country to issue recommendations dedicated to the whole obstetric population and invited pregnant women earlier in the vaccination campaign compared to non-pregnant women of childbearing age. This might have provided a sense of security and confidence in the COVID-19 vaccine among pregnant Belgian women, as well as the opportunity to be vaccinated, explaining the observed high prevalence of vaccinees or women willing to be vaccinated in this country in our cohort. Belgian women were also more likely to answer that they have “absolute trust” in their government and health authorities than the women in the other countries, except for Norway, which had the highest trust in government and health authorities of all countries (Supplementary Figure S2).

Surprisingly, in our survey, being in the third trimester of pregnancy was negatively associated with vaccine willingness. We would have expected the opposite as some national guidelines recommended vaccination with a preference for the 2nd and 3rd trimesters, because COVID-19 infection increases the risk of severe illness/complications especially in the third trimester and because the third trimester was associated with vaccine willingness in the literature [8,10]. One possible explanation regarding this finding could be that women in their third trimester were closer to delivery and thought they had less time to potentially become infected.

Our findings on the specific reasons to believe that COVID-19 vaccines are not effective and/or not safe provide grounds for vaccine hesitancy. This study shows that obstetric populations had inaccurate beliefs regarding the impacts of COVID-19 infection and vaccination during the third wave of the pandemic (e.g., among respondents who believed that COVID-19 vaccines are not (entirely) safe during pregnancy or breastfeeding, 77.33% of pregnant women believed that it could lead to a miscarriage or stillbirth, while 60.1% of pregnant women and 35.5% of breastfeeding women believed that it could cause DNA alterations). This information could help develop protocols to improve the risk perception of COVID-19 vaccines and potentially has wider applicability to other vaccines recommended during pregnancy. To overcome vaccination hesitancy, it is strongly recommended that HCPs inform their patients about the growing evidence of COVID-19 vaccines’ safety. The different sources that pregnant and breastfeeding women trust to provide information about the coronavirus may differ across the five European countries. This information may elucidate the optimal channels to communicate available safety evidence to target populations in different countries.

### 4.2. Strengths and Limitations

The main strength of our study is its international design and uniform data collection across the five countries, while recruiting a high number of participants. Moreover, we collected both descriptive and more in-depth insights on the topic of COVID-19 vaccine hesitancy in a high-risk population and examined how to deal with it. This study was performed with support from national experts of teratology, who aided this research through their previous experience of conducting a multinational study on COVID-19 vaccine willingness among pregnant and breastfeeding women in 2020 [17]. Finally, we also explored the issue of willingness to be vaccinated against COVID-19 in the obstetric population by asking women their opinions and beliefs about the infection, the vaccine, the management of the pandemic, and reliable sources of information about the coronavirus.

However, this study also has some limitations. Nearly 7% of women did not answer the question about COVID-19 vaccination status or willingness to be vaccinated. It is likely that the participants did not answer this question because it was situated towards the end of both questionnaires. An additional possible explanation for women abandoning the survey is linked to the question asked just prior the COVID-19 vaccination/willingness. This question assessed whether a family member had refused the COVID-19 vaccine or not. Multiple participants completely stopped the survey at this question, which might have been perceived as a highly sensitive topic. The women who did not answer the questions about the COVID-19 vaccination/willingness may have not been in favor of vaccination and their drop-out may have biased the results towards overestimating the prevalence of vaccine willingness in our study population.

Our study population does not equally represent all pregnant and postpartum women living in the studied countries [15,16]. Indeed, a higher proportion of respondents who answered the survey were from Norway (74.4% of pregnant women and 67.1% of postpartum women), women with a high educational level (73.8% and 71.1%), and healthcare workers (26.6% and 27.0%). This overrepresentation of women with high educational level and healthcare workers might have led to an overestimation of the prevalence of COVID-19 vaccination status or willingness. On the other hand, the high proportion of Norwegian women who were the least willing to be vaccinated might have led to an underestimation of the prevalence of vaccination status or willingness. Nevertheless, the determinants regarding education and profession were associated with vaccine willingness in univariate analyses but not in the multivariable model. Finally, the timing of the vaccination campaigns and vaccine recommendations differed across countries, which likely had an impact on the composite outcome. The first sensitivity analysis focusing on the willingness to be vaccinated regardless of vaccination status, showed comparable results.

Regarding external generalizability of the findings, our European survey included only high-income countries with a large proportion of women with a high socio-professional background. Consequently, our findings may only be generalizable to high-income countries. Yet, the meta-analysis published by Bhattacharya et al. found that high-income countries, such as Switzerland and Singapore, had lower COVID-19 vaccine acceptance [12]. Despite higher expected vaccination rates in countries with higher gross domestic product per capita, countries such as Norway and Switzerland had a lower vaccination uptake rate than expected. This could reflect country-specific individual concerns that are not related to medical facilities [36]. Moreover, the ideology of healthism and low perceptions of the threat of vaccine-preventable diseases may explain the positive link between socioeconomic status and vaccine hesitancy in high-income countries [37].

Finally, this cross-sectional survey only provides one point estimate during the third wave of the pandemic in 2021, whilst COVID-19 vaccine hesitancy rates among pregnant and breastfeeding women evolved over time (40.0%, 58.0%, and 38.1% in surveys conducted in 2020 and the first and second halves of 2021, respectively) [8]. The willingness to vaccinate is likely to continue to evolve as it relates to (i) the impact of SARS-CoV-2 variants on maternal and pregnancy outcomes, as less severe maternal disease-causing variants emerge, and (ii) the effectiveness of the vaccine against the new variants of concern, both of which evolve over time [1,38].

## 5. Conclusions

COVID-19 vaccine hesitancy among pregnant and postpartum women depends on both on medical history and especially on the opinion that the vaccine is safe during pregnancy and breastfeeding and on the country of residence. Clear public information and clinical guidelines stressing the benefits of vaccination for both mother and child, as well as the absence of the excess risk of vaccination during pregnancy and breastfeeding, are essential to ensure COVID-19 vaccine willingness and uptake in this population.

## Figures and Tables

**Table 1:** Characteristics of the pregnant and postpartum women who participated in the survey and who were included in the analysis on vaccine willingness.

Characteristics		Pregnant Women N = 3194 N (%)	Postpartum Women N = 1659 N (%)
**Country**	Belgium	298 (9.3)	196 (11.8)
	Norway	2376 (74.4)	1113 (67.1)
	Switzerland	165 (5.2)	140 (8.4)
	The Netherlands	123 (3.9)	124 (7.5)
	United Kingdom	232 (7.3)	86 (5.2)
**Maternal age (years)**	18–25	224 (7.0)	94 (5.7)
	26–30	1150 (36.0)	581 (35.0)
	31–35	1301 (40.7)	684 (41.2)
	36–40	406 (12.7)	213 (12.8)
	>40	68 (2.1)	44 (2.7)
	*Missing data*	*45 (1.4)*	*183 (2.6)*
**Relationship** **status**	Partner	3092 (96.8)	1595 (96.1)
	No partner	57 (1.8)	21 (1.3)
	*Missing data*	*45 (1.4)*	*43 (2.6)*
**Professional ** status *	Inactive	348 (10.9)	176 (9.8)
	Active	2799 (87.6)	1430 (79.5)
	*Missing data*	*47 (1.5)*	*53 (3.2)*
**Working in ** **healthcare**	No	1879 (58.8)	947 (57.1)
	Yes	906 (28.8)	486 (29.3)
	*Missing data*	*409 (12.8)*	*226 (13.6)*
**Educational ** Level **	Low	73 (2.3)	34 (2.0)
	Medium	540 (16.9)	290 (17.5)
	High	2516 (78.7)	1279 (77.1)
	*Missing data*	*65 (2.0)*	*56 (3.4)*
**Smoking during ** pregnancy ***	Yes	43 (1.3)	19 (1.1)
	No	3106 (97.2)	1597 (88.8)
	*Missing data*	*45 (1.4)*	*43 (2.6)*
**History of positive SARS-CoV-2 test**	Yes	142 (4.4)	83 (5.0)
	No	3051 (95.5)	1576 (95.0)
	*Missing data*	*1 (0.0)*	*0 (0.0)*
**Chronic illness requiring medication**	Yes	1174 (36.8)	470 (26.1)
	No	1416 (44.3)	790 (43.9)
	*Missing data*	*604 (18.9)*	*399 (24.1)*
**Gravidity**	Primigravida	1607 (50.3)	NA
	Multigravida	1587 (49.7)	NA
**Gestational ** **trimester**	1st trimester	351 (11.0)	NA
	2nd trimester	1102 (34.5)	NA
	3rd trimester	1741 (54.5)	NA
**Breastfeeding at the** **time of survey completion**	Yes	NA	1523 (91.8)
	No	NA	136 (8.2)
**Participated in the COVID-19 survey in 2020 #**	Yes	47 (1.5)	18 (1.1)
	No	3032 (94.9)	1574 (94.9)
	Can’t remember	105 (3.3)	67 (4.0)

Results are expressed as absolute numbers (%). NA = question was not applicable. * At the time of survey completion or before maternity leave. ** highest level of education according to national definitions: low (primary school), medium (high school), and high (beyond high school). *** Smoking during current/previous pregnancy for pregnant and post-partum women, respectively. # A survey on COVID-19 vaccination willingness in pregnant and breastfeeding women was already performed by our team in 2020 during the first wave of the pandemic [17].

**Table 2:** COVID-19 vaccination status and vaccine willingness among pregnant and postpartum women according to country of residence.

Country	Total N (%)		Belgium N (%)		Norway N (%)		Switzerland N (%)		The Netherlands N (%)		United Kingdom N (%)	
Status	Pregnancy	Postpartum	Pregnancy	Postpartum	Pregnancy	Postpartum	Pregnancy	Postpartum	Pregnancy	Postpartum	Pregnancy	Postpartum
Already vaccinated against COVID-19												
Yes	752 (23.5)	662 (39.9)	216 (72.5)	112 (57.1)	268 (11.3)	365 (32.8)	37 (22.4)	56 (40.0)	59 (48.0)	62 (50.0)	172 (74.1)	67 (77.9)
No	2442 (76.5)	997 (60.1)	82 (27.5)	84 (42.9)	2108 (88.7)	748 (67.2)	128 (77.6)	84 (60.0)	64 (52.0)	62 (50.0)	60 (25.9)	19 (22.1)
Unvaccinated women who would be vaccinated against COVID-19 if they had had the opportunity												
Yes	311 (12.7)	654 (65.6)	24 (29.3)	56 (71.8)	242 (11.5)	537 (71.8)	16 (12.5)	26 (31.0)	18 (28.1)	28 (45.2)	11 (18.3)	7 (36.8)
No	1521 (62.3)	156 (15.6)	42 (51.2)	11 (11.5)	1326 (62.9)	86 (11.5)	82 (64.1)	40 (47.6)	36 (56.2)	15 (24.2)	35 (58.3)	4 (21.1)
Do not know	610 (25.0)	187 (18.8)	16 (19.5)	17 (20.2)	540 (25.6)	125 (16.7)	30 (23.4)	18 (21.4)	10 (15.6)	19 (30.6)	14 (23.3)	8 (42.1)
Composite variable: “already vaccinated or willing to get vaccinated against COVID-19”												
Yes	1063 (33.3)	1316 (79.3)	240 (80.5)	168 (85.7)	510 (21.5)	902 (81.0)	53 (32.1)	82 (58.6)	77 (62.6)	90 (72.6)	183 (78.9)	74 (86.0)
No	2131 (66.7)	343 (20.7)	58 (19.5)	28 (14.3)	1866 (78.5)	211 (19.0)	112 (67.9)	58 (41.4)	46 (37.4)	34 (27.4)	49 (21.1)	12 (14.0)

Results are expressed as absolute numbers (%).

**Table 3:** Specific reasons to believe that COVID-19 vaccines are not effective and/or not safe among pregnant and postpartum women, stratified by breastfeeding status.

	Pregnant Women				Postpartum Women	
	All N (%)	1st Trimester N (%)	2nd Trimester N (%)	3rd Trimester N (%)	Breastfeeding N (%)	No Breastfeeding N (%)
“Please indicate why you believe COVID-19 vaccines are not (entirely) safe during pregnancy, breastfeeding or postpartum. You can indicate multiple reasons.” *						
“I believe that the long-term effects of these vaccines are not known yet”	1908/1958 (97.4)	202/208 (97.1)	653/69 (97.6)	1053/1081 (97.4)	651/726 (89.7)	61/189 (32.3)
“I believe that some steps of the usual process of vaccine development and approval were not fully completed or bypassed”	917/1030 (89.0)	87/99 (87.9)	328/369 (88.9)	502/562 (89.3)	313/417 (75.1)	36/171 (21.0)
“I believe that it bears a potential risk for my unborn child’s growth and development”	761/872 (87.2)	105/118 (89)	302/343 (88)	354/411 (86.1)	NA	NA
“I believe that it bears a potential risk of malformation for my unborn”	548/671 (81.7)	79/91 (86.8)	221/268 (82.5)	248/312 (79.5)	NA	NA
“I believe that it bears potential risks for my own health”	431/552 (78.1)	54/65 (83.1)	151/201 (75.1)	226/286 (79)	216/327 (66.1)	22/161 (13.7)
“I believe that it could lead to a miscarriage or stillbirth”	470/608 (77.3)	80/194 (85.1)	212/265 (80)	178/249 (71.5)	NA	NA
“Other reason”	399/551 (72.4)	39/55 (70.9)	136/204 (66.7)	224/292 (76.7)	70/205 (34.2)	6/144 (4.2)
“I believe that other non-medical treatments for COVID-19 may be safer”	305/464 (65.7)	26/42 (61.9)	97/164 (59.1)	182/258 (70.5)	37/174 (21.3)	3/143 (2.1)
“I believe that it could negatively affect my fertility”	235/388 (60.6)	36/50 (72)	88/155 (56.8)	111/183 (60.7)	104/230 (45.2)	10/147 (6.8)
“I believe that it could cause DNA alterations”	229/381 (60.1)	35/50 (70)	92/154 (59.7)	102/177 (57.6)	72/203 (35.5)	6/145 (4.1)
“I believe that other medical treatments for COVID-19 may be safer”	184/341 (54.0)	18/35 (51.4)	64/130 (49.2)	102/176 (58)	26/163 (16.0)	4/144 (2.8)
“I could catch (/transmit) the disease through the vaccine”	54/214 (25.2)	105/118 (89)	302/343 (88)	354/411 (86.1)	24/156 (15.4)	5/144 (3.5)
“I generally do not believe vaccines are safe”	29/190 (15.3)	4/21 (19)	13/82 (15.9)	12/87 (13.8)	17/153 (11.1)	11/142 (7.7)
“I believe that it bears potential risks for my nursing infant”	NA	NA	NA	NA	273/372 (73.4)	NA

* This multiple-choice question was provided only to women who believed that COVID-19 vaccines are either “safe” or “not very safe” or “not safe at all”, but not to those who believed that the vaccines are “very safe” or “extremely safe”. The reasons are listed in descending order with respect to pregnant women’s responses. The denominators are different for each question, because the women did not answer to all questions of the survey.

## Data Availability

The collected data are presented in the manuscript and in the Supplementary Materials.

## Supplementary Materials

The following supporting information can be downloaded at: https://www.mdpi.com/article/10.3390/v15051090/s1, Supplementary Material S1: English version of the survey relevant to this manuscript; Supplementary Material S2: Recruitment tools utilized and internet penetration rates; Supplementary Material S3: Rollout of vaccination against COVID-19 in pregnant women and vaccination rates in the adult population in the five participating countries as of 1 July 2021; Supplementary Material S4: Context of pandemic and rollout of vaccination against COVID-19 in pregnant women in the five participating countries as of 1 July 2021; Supplementary Table S1: Characteristics of the pregnant and postpartum women who participated in the survey but were excluded because they did not answer the question on vaccination status; Supplementary Table S2: Association between socio-demographic, medical, obstetrical and ideological characteristics, and COVID-19 vaccination or willingness to be vaccinated among pregnant and postpartum women; Supplementary Table S3: Association between socio-demographic, medical, obstetrical and ideological characteristics, and COVID-19 vaccine willingness, whatever the vaccination status, among pregnant women; Supplementary Table S4: Association between socio-demographic, history of COVID-19, medical and obstetrical characteristics, and COVID-19 vaccination or willingness to be vaccinated among breastfeeding women; Supplementary Table S5: Specific reasons to believe that COVID-19 vaccines are not effective and/or not safe among pregnant and postpartum women, stratified by breastfeeding; Supplementary Figure S1: Perceptions on the safety and effectiveness of conventional and COVID-19 vaccines among pregnant and postpartum women, vaccinated or willing to be vaccinated or not; Supplementary Figure S2: Sources trusted to provide information regarding the coronavirus by obstetrical status and by country; Supplementary Figure S3: Hierarchical cluster analysis of determinants associated with vaccination adherence among pregnant and postpartum women.

## References

[1] Favre G., Maisonneuve E., Pomar L., Daire C., Poncelet C., Quibel T., Monod C., Martinez de Tejada B., Schaffer L., Papadia A. (2023). Maternal and perinatal outcomes following pre-Delta, Delta, and Omicron SARS-CoV-2 variants infection among unvaccinated pregnant women in France and Switzerland: A prospective cohort study using the COVI-PREG registry. Lancet Reg. Health Eur..

[2] Smith E.R., Oakley E., Grandner G.W., Rukundo G., Farooq F., Ferguson K., Baumann S., Waldorf K.M.A., Afshar Y., Ahlberg M. (2023). Clinical risk factors of adverse outcomes among women with COVID-19 in the pregnancy and postpartum period: A sequential, prospective meta-analysis. Am. J. Obstet. Gynecol..

[3] Lassi Z.S., Ali A., Das J.K., Salam R.A., Padhani Z.A., Irfan O., Bhutta Z.A. (2021). A systematic review and meta-analysis of data on pregnant women with confirmed COVID-19: Clinical presentation, and pregnancy and perinatal outcomes based on COVID-19 severity. J. Glob. Health.

[4] Prasad S., Kalafat E., Blakeway H., Townsend R., O’Brien P., Morris E., Draycott T., Thangaratinam S., Le Doare K., Ladhani S. (2022). Systematic review and meta-analysis of the effectiveness and perinatal outcomes of COVID-19 vaccination in pregnancy. Nat. Commun..

[5] Halasa N.B., Olson S.M., Staat M.A., Newhams M.M., Price A.M., Pannaraj P.S., Boom J.A., Sahni L.C., Chiotos K., Cameron M.A. (2022). Maternal Vaccination and Risk of Hospitalization for COVID-19 among Infants. N. Engl. J. Med..

[6] Low J.M., Gu Y., Ng M.S.F., Wang L.W., Amin Z., Zhong Y., MacAry P.A. (2022). Human Milk Antibodies after BNT162b2 Vaccination Exhibit Reduced Binding against SARS-CoV-2 Variants of Concern. Vaccines.

[7] Shook L.L., Edlow A.G. (2023). Safety and Efficacy of Coronavirus Disease 2019 (COVID-19) mRNA Vaccines During Lactation. Obstet. Gynecol..

[8] Bianchi F.P., Stefanizzi P., Di Gioia M.C., Brescia N., Lattanzio S., Tafuri S. (2022). COVID-19 vaccination hesitancy in pregnant and breastfeeding women and strategies to increase vaccination compliance: A systematic review and meta-analysis. Expert Rev. Vaccines.

[9] Shamshirsaz A.A., Hessami K., Morain S., Afshar Y., Nassr A.A., Arian S.E., Asl N.M., Aagaard K. (2022). Intention to Receive COVID-19 Vaccine during Pregnancy: A Systematic Review and Meta-analysis. Am. J. Perinatol..

[10] Nindrea R.D., Djanas D., Warsiti, Darma I.Y., Hendriyani H., Sari N.P. (2022). The risk factors and pregnant women’s willingness toward the SARS-CoV-2 vaccination in various countries: A systematic review and meta-analysis. Clin. Epidemiol. Glob. Health.

[11] Azami M., Nasirkandy M.P., Esmaeili Gouvarchin Ghaleh H., Ranjbar R. (2022). COVID-19 vaccine acceptance among pregnant women worldwide: A systematic review and meta-analysis. PLoS ONE.

[12] Bhattacharya O., Siddiquea B.N., Shetty A., Afroz A., Billah B. (2022). COVID-19 vaccine hesitancy among pregnant women: A systematic review and meta-analysis. BMJ Open.

[13] Badell M.L., Dude C.M., Rasmussen S.A., Jamieson D.J. (2022). COVID-19 vaccination in pregnancy. BMJ.

[14] Gerbier E., Favre G., Tauqeer F., Winterfeld U., Stojanov M., Oliver A., Passier A., Nordeng H., Pomar L., Baud D. (2022). Self-Reported Medication Use among Pregnant and Postpartum Women during the Third Wave of the COVID-19 Pandemic: A European Multinational Cross-Sectional Study. Int. J. Environ. Res. Public Health.

[15] Tauqeer F., Ceulemans M., Gerbier E., Passier A., Oliver A., Foulon V., Panchaud A., Lupattelli A., Nordeng H. (2023). Mental health of pregnant and postpartum women during the third wave of the COVID-19 pandemic: A European cross-sectional study. BMJ Open.

[16] Araya R.A., Tauqeer F., Ceulemans M., Gerbier E., Maisonneuve E., Passier A., Oliver A., Panchaud A., Lupattelli A., Nordeng H. (2023). Pregnancy- and Birth-Related Experiences among Postpartum Women during the Third Wave of the COVID-19 Pandemic—A Multinational European Study. Pharmacoepidemiology.

[17] Ceulemans M., Foulon V., Panchaud A., Winterfeld U., Pomar L., Lambelet V., Cleary B., O’Shaughnessy F., Passier A., Richardson J.L. (2021). Vaccine Willingness and Impact of the COVID-19 Pandemic on Women’s Perinatal Experiences and Practices-A Multinational, Cross-Sectional Study Covering the First Wave of the Pandemic. Int. J. Environ. Res. Public Health.

[18] Ceulemans M., Foulon V., Ngo E., Panchaud A., Winterfeld U., Pomar L., Lambelet V., Cleary B., O’Shaughnessy F., Passier A. (2021). Mental health status of pregnant and breastfeeding women during the COVID-19 pandemic-A multinational cross-sectional study. Acta Obstet. Gynecol. Scand..

[19] Superior Health Council Belgium Recommendations for the Vaccination against SARS-CoV-2 with Messenger RNA Vaccines of Pregnant Women, Women Willing to Get Pregnant and Breastfeeding Women. 21 May 2021. https://www.health.belgium.be/sites/default/files/uploads/fields/fpshealth_theme_file/20210623_hgr-9622_vaccinatie_zwangere_en_lacterende_vrouwen_vweb.pdf.

[20] Norwegian Institute of Public Health Advice and Information for Women Who Are Pregnant or Breastfeeding, Published 14 March 2020, Updated 25 March 2022. https://www.fhi.no/en/op/novel-coronavirus-facts-advice/facts-and-general-advice/advice-and-information-for-pregnant-women/#vaccination-of-pregnant-women.

[21] Federal Office of Public Health Recommendations of Federal Office of Public Health on Coronavirus Vaccination in Pregnant Women. https://www.bag.admin.ch/bag/en/home/krankheiten/ausbrueche-epidemien-pandemien/aktuelle-ausbrueche-epidemien/novel-cov/impfen.html#-1430125092.

[22] Government of The Netherlands, National Institute for Public Health and the Environment Pregnancy and the COVID-19 Vaccine. https://www.government.nl/topics/coronavirus-covid-19/dutch-vaccination-programme/safety-and-development-of-vaccines.

[23] Royal College of Obstetricians and Gynecologists COVID-19 Vaccines, Pregnancy and Breastfeeding. https://www.rcog.org.uk/guidance/coronavirus-covid-19-pregnancy-and-women-s-health/vaccination/covid-19-vaccines-pregnancy-and-breastfeeding-faqs/.

[24] Our World in Data COVID-19 Data Explorer. https://ourworldindata.org/covid-vaccinations?country=OWID_WRL.

[25] Ortqvist A.K., Dahlqwist E., Magnus M.C., Ljung R., Jonsson J., Aronsson B., Pasternak B., Haberg S.E., Stephansson O. (2022). COVID-19 vaccination in pregnant women in Sweden and Norway. Vaccine.

[26] National Institute for Public Health and the Environment Archief Wekelijkse Update Vaccinatiecijfers July 2021. https://www.rivm.nl/covid-19-vaccinatie/archief-wekelijkse-update-vaccinatiecijfers-2021.

[27] Zhang Z., Murtagh F., Van Poucke S., Lin S., Lan P. (2017). Hierarchical cluster analysis in clinical research with heterogeneous study population: Highlighting its visualization with R. Ann. Transl. Med..

[28] von Elm E., Altman D.G., Egger M., Pocock S.J., Gotzsche P.C., Vandenbroucke J.P., Initiative S. (2014). The Strengthening the Reporting of Observational Studies in Epidemiology (STROBE) Statement: Guidelines for reporting observational studies. Int. J. Surg..

[29] Su X., Lu H., Li X., Luo M., Li F., Zhang Q. (2022). COVID-19 vaccine hesitancy in periconceptional and lactating women: A systematic review and meta-analysis protocol. BMJ Open.

[30] Schaal N.K., Zollkau J., Hepp P., Fehm T., Hagenbeck C. (2022). Pregnant and breastfeeding women’s attitudes and fears regarding the COVID-19 vaccination. Arch. Gynecol. Obstet..

[31] Fajar J.K., Sallam M., Soegiarto G., Sugiri Y.J., Anshory M., Wulandari L., Kosasih S.A.P., Ilmawan M., Kusnaeni K., Fikri M. (2022). Global Prevalence and Potential Influencing Factors of COVID-19 Vaccination Hesitancy: A Meta-Analysis. Vaccines.

[32] Egloff C., Couffignal C., Cordier A.G., Deruelle P., Sibiude J., Anselem O., Benachi A., Luton D., Mandelbrot L., Vauloup-Fellous C. (2022). Pregnant women’s perceptions of the COVID-19 vaccine: A French survey. PLoS ONE.

[33] Marban-Castro E., Nedic I., Ferrari M., Crespo-Mirasol E., Ferrer L., Noya B., Marin A., Fumado V., Lopez M., Menendez C. (2022). Perceptions of COVID-19 Maternal Vaccination among Pregnant Women and Healthcare Workers and Factors That Influence Vaccine Acceptance: A Cross-Sectional Study in Barcelona, Spain. Vaccines.

[34] Mannocci A., Scaglione C., Casella G., Lanzone A., La Torre G. (2022). COVID-19 in Pregnancy: Knowledge about the Vaccine and the Effect of the Virus. Reliability and Results of the MAMA-19 Questionnaire. Int. J. Environ. Res. Public Health.

[35] Jennings W., Stoker G., Bunting H., Valgarethsson V.O., Gaskell J., Devine D., McKay L., Mills M.C. (2021). Lack of Trust, Conspiracy Beliefs, and Social Media Use Predict COVID-19 Vaccine Hesitancy. Vaccines.

[36] Roghani A. (2021). The relationship between macro-socioeconomics determinants and COVID-19 vaccine distribution. AIMS Public Health.

[37] Kirbiš A. (2023). The Impact of Socioeconomic Status, Perceived Threat and Healthism on Vaccine Hesitancy. Sustainability.

[38] Villar J., Soto Conti C.P., Gunier R.B., Ariff S., Craik R., Cavoretto P.I., Rauch S., Gandino S., Nieto R., Winsey A. (2023). Pregnancy outcomes and vaccine effectiveness during the period of omicron as the variant of concern, INTERCOVID-2022: A multinational, observational study. Lancet.

