# Supplementary material for: Determinants of Vaccination and Willingness to Vaccinate against COVID-19 among Pregnant and Postpartum Women during the Third Wave of the Pandemic: A European Multinational Cross-Sectional Survey
Source: Viruses. 2023 Apr 29;15(5):1090. doi: 10.3390/v15051090 (PMC10220755; doi:10.3390/v15051090)
Supplement: Supplementary file 1 [file viruses-15-01090-s001.zip › viruses-2343220-supplementary.pdf]

Supplementary Materials

Supplementary Material S1: English version of the survey relevant to this manuscript
Supplementary Material S2: Recruitment tools utilized and internet penetration rates
Supplementary Material S3: Rollout of vaccination against COVID in pregnant women and vaccination rates in the adult population in the five participating countries as of July 1st, 2021
Supplementary Material S4: Context of pandemic and rollout of vaccination against COVID in pregnant women in the five participating countries as of July 1st, 2021

Supplementary Table S1: Characteristics of the pregnant and postpartum women participating in the survey, but excluded because they did not answer the question on vaccination status
Supplementary Table S2: Association between socio-demographic, medical, obstetrical and ideological characteristics, and COVID-19 vaccination or willingness to get vaccinated among pregnant and postpartum women
Supplementary Table S3: Association between socio-demographic, medical, obstetrical and ideological characteristics, and COVID-19 vaccine willingness, whatever the vaccination status, among pregnant women
Supplementary Table S4: Association between socio-demographic, history of COVID-19, medical and obstetrical characteristics, and COVID-19 vaccination or willingness to get vaccinated among breastfeeding women
Supplementary Table S5: Specific reasons to believe that COVID vaccines are not effective and/or not safe among pregnant and postpartum women, stratified by breastfeeding

Supplementary Figure S1: Perceptions on the safety and effectiveness of conventional and COVID-19 vaccines among pregnant and postpartum women, vaccinated or willing to get vaccinated or not.
Supplementary Figure S2: Sources trusted to provide information regarding the coronavirus by obstetrical status and by country
Supplementary Figure S3: Hierarchical cluster analysis of determinants associated with vaccination adherence among pregnant and postpartum women

Supplementary Material S1: English version of the questionnaire relevant to this manuscript.

Did you participate in the previous COVID-survey distributed in June-July 2020?
a. Yes / b. No / c. I don't know

A. Pregnancy-specific questionnaire

- How many weeks pregnant are you right now? (dropdown menu: 0-42)

- Was the pregnancy planned?
  a. Yes / b. No / c. No, but it was not unexpected

- Have you been pregnant before?
  a. Yes / b. No

- If yes to previous question: How many children do you already have? (not including the current pregnancy)

B. Postpartum-specific questionnaire

Are you currently breastfeeding and/or giving expressed milk?
a. Yes / b. No

C. Coronavirus infection (pregnancy and postpartum questionnaires)

- Did you have a positive test for coronavirus infection?
  a. Yes / b. No

- If no to previous question: Do you believe you were infected with the coronavirus although not confirmed by a test?
  a. Yes / b. No

- Has any member(s) of your household or close family tested positive for the coronavirus since the start of the pandemic?
  a. Yes / b. No

D. Covid-19 and disease severity (pregnancy and postpartum questionnaires)

- If yes to previous questions on personal COVID infection: Can you specify the severity of the COVID-19 disease that affected you (multiple answers possible):
  a. I have/ had no or mild symptoms
  b. I have/ had moderate symptoms
  c. I have/ had long-term symptoms of the disease
  d. I was hospitalized but not in intensive care unit (ICU)
  e. I was hospitalized in ICU

- If yes to previous question on familial COVID infection: Please indicate which applies regarding the severity of COVID-19 among the member(s) of your household / close family (multiple answers possible):

a. passed away
b. hospitalized in intensive care unit (ICU)
c. hospitalized but not in ICU
d. long-term symptoms of the disease
e. mild to no symptoms
f. moderate symptoms

E. Covid-19 and pregnancy (pregnancy questionnaire)

- Do you believe coronavirus infection can be more severe during pregnancy than among non-pregnant women of the same age?
    a. Yes / b. No

- If yes to previous question: can you specify why you believe a coronavirus infection can be more severe during pregnancy (multiple answers possible):
    a. I believe pregnant women are at increased risk of more severe disease with coronavirus infection (for example intensive care unit admission) due to the underlying pregnancy state
    b. I believe coronavirus infection during pregnancy can cause malformations to the unborn child
    c. I believe coronavirus infection during pregnancy can affect the growth and development of the unborn child
    d. I believe coronavirus infection during pregnancy increases the risk of pregnancy complications (for example preterm birth)
    e. I believe coronavirus infection could be transmitted to my baby during delivery
    f. Other reason: ….

F. COVID-19 and breastfeeding (postpartum questionnaire)

- To what extent do you believe that breastfeeding can be risky for the nursing infant if a breastfeeding mother is infected with coronavirus?
    a. Extremely risky
    b. Very risky
    c. Risky
    d. Not very risky
    e. Not risky at all

- If a, b or c to previous question: Can you specify why you believe that breastfeeding can be risky for the nursing infant if a breastfeeding mother is infected with the coronavirus (multiple answers possible):
    a. I believe the virus can be transmitted to the baby through milk
    b. I believe the baby could be infected while breastfeeding (through close contact)
    c. I believe the disease could stop the mother from breastfeeding (feeling too sick)
    d. I believe the disease could negatively affect the milk production
    e. Other

G. Coronavirus and restrictive measures (pregnancy and postpartum questionnaires)

Please rate, on a scale from 1 to 5, how useful you believe the following measures are to prevent coronavirus transmission (1= Not at all useful, 2= Not very useful, 3= Useful, 4= Very useful, 5= Extremely useful):

a. Using disinfectant gel/hand washing regularly
b. Wearing masks
c. Keeping a social distance
d. Working from home
e. Curfew
f. A (semi-) Lockdown
g. Vaccination

H. Use of vaccines

Conventional vaccines perception (pregnancy and postpartum questionnaires)

- To which extent do you believe conventional vaccines are **safe in the general population**?
    a. Extremely safe
    b. Very safe
    c. Safe
    d. Not very safe
    e. Not safe at all

- To which extent do you believe conventional vaccines are **effective in the general population**?

a. Extremely effective
b. Very effective
c. Effective
d. Not very effective
e. Not effective at all

Conventional pregnancy vaccines perception (Pregnancy questionnaire)

- To which extent do you believe vaccines that are recommended **during pregnancy are safe** for yourself and your unborn child (influenza, pertussis)?
  a. Extremely safe
  b. Very safe
  c. Safe
  d. Not very safe
  e. Not safe at all

- To which extent do you believe vaccines that are recommended **during pregnancy are effective** to protect yourself and your unborn child (influenza, pertussis)?
  a. Extremely effective
  b. Very effective
  c. Effective
  d. Not very effective
  e. Not effective at all

Conventional vaccines perception (Postpartum questionnaire)

To which extent do you believe conventional vaccines during breastfeeding **are safe** for the mother and her nursing infant?
a. Extremely safe
b. Very safe
c. Safe
d. Not very safe
e. Not safe at all

Flu vaccination (pregnancy and postpartum questionnaires)

- Did you get a flu vaccination last winter (2020-2021)?
  a. Yes / b. No

- If no to previous question: Why did you not get a flu vaccination last winter (2020-2021)? (multiple answers possible)
  a. It was not suggested by a healthcare professional
  b. I was unsure whether it was safe for my unborn child
  c. I thought that I didn't need it as a pregnant woman
  d. I generally refuse getting vaccines
  e. I was advised by a friend/family member/ close relation not to do it
  f. Other reason: ….

- If yes to breastfeeding: Have you already been vaccinated against coronavirus?
  a. Yes / b. No

- If no to previous question: Indicate which applies to you: "I would get vaccinated against coronavirus while breastfeeding if I had the opportunity."
  a. Yes / b. No / c. I don't know

- If no to breastfeeding: Have you already been vaccinated against coronavirus?
  a. Yes / b. No

- If no to previous question: Indicate which applies to you: "I would get vaccinated against coronavirus if I had the opportunity."
    a. Yes / b. No / c. I don't know

Covid-19 vaccine perception (pregnancy and postpartum questionnaires)

- To which extent do you believe COVID-19 vaccines are **effective** to protect the general population?
    a. Extremely effective
    b. Very effective
    c. Effective
    d. Not very effective
    e. Not effective at all

- To which extent do you believe COVID-19 vaccines are **safe** in the general population?
    a. Extremely safe
    b. Very safe
    c. Safe
    d. Not very safe
    e. Not safe at all

Covid-19 vaccine perception (Pregnancy questionnaire)

- To which extent do you believe COVID-19 vaccines are **effective** to protect yourself and your unborn child during pregnancy?
    a. Extremely effective
    b. Very effective
    c. Effective
    d. Not very effective
    e. Not effective at all

- To which extent do you believe COVID-19 vaccines are **safe** for yourself and your unborn child during pregnancy?
    a. Extremely safe
    b. Very safe
    c. Safe
    d. Not very safe
    e. Not safe at all

- If answers c, d or e to previous question then: Please select the reason(s) you believe COVID-19 vaccines are not (entirely) **safe during pregnancy** (multiple answers possible):
    a. I believe that it bears potential risks for my own health
    b. I believe that it bears a potential risk of malformation for my unborn child
    c. I believe that it bears a potential risk for my unborn child's growth and development
    d. I believe that I could catch the disease through the vaccine
    e. I believe that it could cause DNA alterations
    f. I believe that it could negatively affect my fertility
    g. I believe that it could lead to a miscarriage or stillbirth
    h. I believe that some steps of the usual process of vaccine development and approval were not fully completed or bypassed
    i. I believe that the long-term effects of these vaccines are not known yet
    j. I believe that other medical treatments for COVID-19 may be safer
    k. I believe that other non-medical treatments for COVID-19 may be safer
    l. I generally don't believe vaccines are safe
    m. Other

Covid-19 vaccine perception (Postpartum questionnaire)

- If answer yes to breastfeeding: To which extent do you believe COVID-19 vaccines are **effective** to protect yourself and your child while breastfeeding?

a. Extremely effective
b. Very effective
c. Effective
d Not very effective
e. Not effective at all

- If answer no to breastfeeding: To which extent do you believe COVID-19 vaccines are **effective** to protect yourself?
  a. Extremely effective
  b. Very effective
  c. Effective
  d. Not very effective
  e. Not effective at all

- If answer yes to breastfeeding: To which extent do you believe COVID-19 vaccines are **safe** for **yourself and your child** while breastfeeding?
  a. Extremely safe
  b. Very safe
  c. Safe
  d. Not very safe
  e. Not safe at all

- If answers c, d or e to previous question then: Please select the reason(s) you believe that COVID-19 vaccines are not (entirely) safe **during breastfeeding** (multiple answers possible).
  a. I believe that it bears potential risks for my own health
  b. I believe that it bears potential risks for my nursing infant
  c. I believe that I could catch/transmit the disease through the vaccine
  d. I believe that it could cause DNA alterations
  e. I believe that it could negatively affect my fertility
  f. I believe that some steps of the usual process of vaccine development and approval were not fully completed or bypassed
  g. I believe that the long-term effects of these vaccines are not known yet
  h. I believe that other medical treatments for COVID-19 may be more safe
  i. I believe that other non-medical treatments for COVID-19 may be more safe
  j. I generally don't believe vaccines are safe
  k. Other

- If answer no to breastfeeding: To which extent do you believe COVID-19 vaccines are **safe** for **yourself**?
  a. Extremely safe
  b. Very safe
  c. Safe
  d. Not very safe
  e. Not safe at all

- If answers c, d or e to previous question then: Please select the reason(s) you believe that COVID-19 vaccines are not (entirely) safe (multiple answers possible):
  a. I believe that it bears potential risks for my own health
  b. I believe that I could catch the disease through the vaccine
  c. I believe that it could cause DNA alterations
  d. I believe that it could negatively affect my fertility
  e. I believe that some steps of the usual process of vaccine development and approval were not fully completed or bypassed
  f. I believe that the long-term effects of these vaccines are not known yet
  g. I believe that other medical treatments for COVID-19 may be more safe
  h. I believe that other non-medical treatments for COVID-19 may be more safe
  i. I generally don't believe vaccines are safe

j. Other

Influence of family / friends (pregnancy and postpartum questionnaires) #

- Has any member of your family or close relation refused a COVID-19 vaccination?
  a. Yes / b. No

- If yes to previous question: Has his/her decision to refuse a COVID-19 vaccine contributed to your perception of COVID-19 vaccination?
  a. Yes / b. No

- Have you already been vaccinated against coronavirus?
  a. Yes / b. No

- If answer no to previous question: Indicate which applies to you: "I would get vaccinated against coronavirus during pregnancy if I had the opportunity."
  a. Yes / b. No / c. I don't know

Use of medicines (pregnancy and postpartum questionnaires) #

- Did you use any medication(s) in the past 3 months?
  a. Yes / b. No

- If answer yes to previous question: Please indicate for which chronic illnesses you have used a medication in the past 3 months? (i.e., chronic illnesses are conditions that already existed before your pregnancy) (multiple answers possible)?
  a. Asthma
  b. Allergy
  c. Cardiovascular disease (including high blood pressure, high cholesterol, heart disease, etc.)
  d. Depression
  e. Diabetes
  f. Epilepsy
  g. Hypothyroidism (underactive thyroid)
  h. Rheumatic diseases (including rheumatoid arthritis, psoriatic arthritis, etc.)
  i. Inflammatory bowel disease (including Crohn's disease, Ulcerative colitis, etc.)
  j. Anxiety
  k. Other: ….
  l. None

Coronavirus and information sources (pregnancy and postpartum questionnaires) #

Please rate how much you trust the following sources of information to provide reliable information regarding the coronavirus (0= Distrust, 1= Very little trust, 2= Little trust, 3= Moderate trust, 4= Great trust, 5= Absolute trust):

a. The government
b. Health authorities
c. General physician
d. Obstetrician
e. Midwife
f. Pharmacist
g. My family /friends
h. The media (newspapers, TV news channels etc.)
i. The internet

Personal background (pregnancy and postpartum questionnaires) #

- Year of birth? (Dropdown menu)

- Do you currently have a partner?

a. Yes / b. No

- Have you smoked since you found out you were pregnant?
  a. Yes / b. No

- What is your highest level of education?
  a. Primary education
  b. Professional secondary education
  c. Technical secondary education
  d. Artistic secondary education
  e. General secondary education
  f. Professional bachelor
  g. Academic bachelor
  h. Master
  i. PhD
  j. Other: ……….

- What is your current professional status? (if you are currently not working because of pregnancy complications or maternity leave, please fill in the professional status you had before)
  a.Employee
  b.Self-employed
  c.Civil service employee
  d.Student
  e. Homemaker
  f. Jobseeker/unemployed
  g. Incapacitated/disabled
  h. Other

- If answer a,b,c,d or h then: Are/were you working/ following an education in healthcare?

- If answer a,b,c or h then: Does your employment put you at increased risk of catching COVID-19 ?

Legend:

The patients excluded from the analysis did not answers the questions including in the sections "Influence of family / friends", "Use of medicines", "Coronavirus and information sources" and "Personal background" which are located at the end of the questionnaire

Supplementary Material S2: Recruitment tools utilized and internet penetration rates

Country	*Websites used for recruitment*	*Internet penetration rates (%)*
Norway	www.altformamma.no (i.e. pregnancy website); several Facebook sites (e.g. Facebook: Gravid i coronatider); pregnancy forums (e.g. Facebook: Termin september 2020); mobile application (Helseappen).	99.0[1]
Belgium	www.gezondzwangerworden.be; several Facebook pages and websites of perinatal organizations, midwives, lactation consultants, community pharmacies,…	96.0[2]
Switzerland	www.chuv.ch; www.swissmom.ch; several Facebook pages;	95.0[3]
The Netherlands	www.consumentenbond.nl; www.24baby.nl; several Facebook sites (e.g. ik ben zwanger, kindje klein); website for midwives (www.deverloskundige.nl); Lareb website (www.lareb.nl and www.lareb.nl/tis-knowledge).	94.2[4]
UK	www.medicinesinpregnancy.org (i.e. the UKTIS patient facing website); Twitter (UKTIS account); Facebook (local maternity/mother and baby groups).	99.3[5]

Sources of internet penetration rates: [1] Statistics Norway (https://www.ssb.no/en); [2] Statistics Flanders, Belgium (www.statistiekvlaanderen.be); [3]Statistics Switzerland (www.bfs.admin.ch); [4]Statistics Netherlands (www.statistica.com); [5] Statistics UK (https://www.ons.gov.uk)

Country	Data Collection period (2021)	Recommendations for vaccination in pregnant women with corresponding start dates	Percentage of the population over 18 years (1 dose/ 2 doses) as of July 1st 2021
Belgium	June 10th to July 18th	High risk of infection*: 21.01.2021<br>At-risk pregnancies: 21.01.2021<br>All pregnant women: 15.04.2021 (irrespective of trimester)<br>Booster dose: 16.12.2021<br>Breastfeeding: permitted on 21.01.2021	64% / 44%
Norway	July 1st to 24th	High risk of infection*: 29.04.2021<br>At-risk pregnancies: 18.08.2021<br>Pregnant women (preferably in the 2nd/3rd trimester,<br>1st trimester for at-risk pregnant women): 18.08.2021<br>Booster dose: 19.01.2022<br>All pregnant women (irrespective of trimester): 31.01.2022<br>Breastfeeding: 25.03.2022	51% / 29%
Switzerland	June 14th to August 22nd	High risk of exposure: 14.04.21<br>At-risk pregnancies in 2nd or 3rd trimester: 14.04.21<br>All pregnant women from 12 weeks (possible in 1st trimester): 14.09.21<br>Booster dose: 26.11.21<br>Breastfeeding: recommended 14.09.21	50% / 38%
The Netherlands	June 11th to July 18th	High risk of exposure: 08.01.2021<br>Comorbidities**: 08.01.2021 (strongly recommended)<br>All pregnant women: 22.04.2021 (should be vaccinated routinely, when they qualify according to the national vaccination strategy; 2nd and 3rd trimesters preferred)<br>Booster dose. 17.12.2021<br>Breastfeeding: permitted	72% / 41%
United Kingdom	June 14th to July 30th	High risk of exposure*: 30.12.2020<br>Comorbidities: 30.12.2020<br>All pregnant women: 16.04.2021<br>- offered to all pregnant women at the same time as the rest of the population based on their age and clinical risk group<br>- vaccination preferred before 3rd trimester<br>- Pregnant women would be placed on the priority list for vaccination: 16.12.2021<br>Booster dose: 16.12.2021<br>Breastfeeding: permitted on 30.12.2020	85% / 63%

Supplementary Material S3: Rollout of vaccination against COVID in pregnant women and vaccination rates in the adult population in the five participating countries as of July 1st, 2021

* healthcare professionals with a high risk of exposure to SARS-CoV-2
** These illnesses include: chronic airway or lung problems who are under treatment, chronic heart patients who are therefore eligible for a flu shot, diabetes who are poorly adjusted and / or with complications, kidney disease requiring dialysis or waiting for one kidney transplant, reduced resistance to infections because of taking medicines for an autoimmune disease and women who have undergone an organ- or stem cell transplantation, blood disorder, reduced resistance because they take immune suppressive medication. Cancer patients during or within three months after chemotherapy and / or radiation, severe immune disorders for which they need treatment from a doctor. Women who don't have a spleen, or a not functioning spleen, human immunodeficiency virus infection, severe liver disease, very seriously overweight women, maternal age over 35 years and/or with migration background.

Supplementary Material S4: Context of pandemic and rollout of vaccination against COVID in general population in the five participating countries as of July 1st, 2021

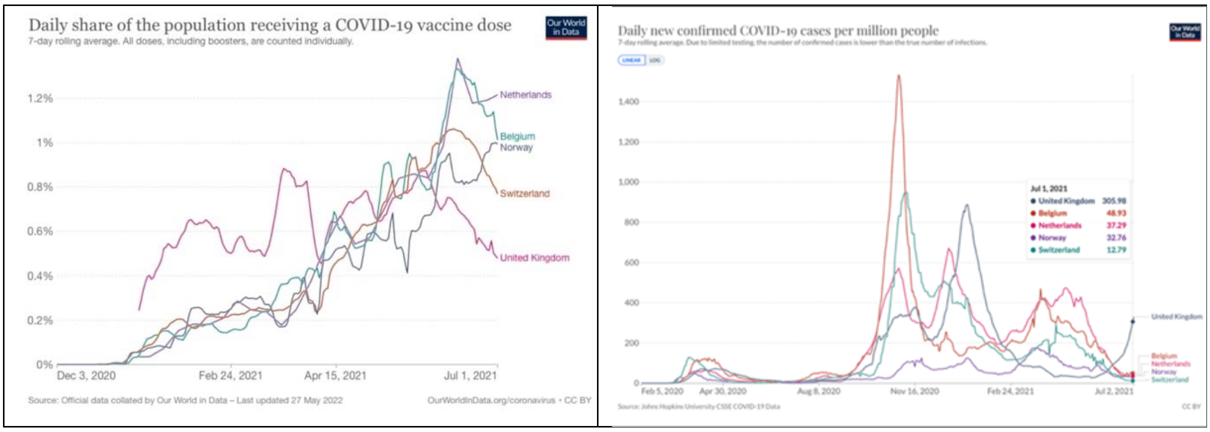

These plots are extracted from the website “Our world in Data” available at https://ourworldindata.org/coronavirus

Supplementary Table S1: Characteristics of the pregnant and postpartum women participating in the survey, but excluded because they did not answer the question on vaccination status

Characteristics		Pregnant women who did not answer to vaccination status N=216 N (%)	Postpartum women who did not answer to vaccination status N=140 N (%)
**Country**	Belgium	62 (28.7)	39 (27.9)
	Norway	0 (0)	0 (0)
	Switzerland	45 (20.8)	36 (25.7)
	Netherlands	52 (24.1)	31 (22.1)
	United Kingdom	57 (26.4)	34 (24.3)
**Maternal age #**	18-25	0 (0)	0 (0)
	26-30	0 (0)	0 (0)
	31-35	0 (0)	0 (0)
	36-40	0 (0)	0 (0)
	>40	0 (0)	0 (0)
	Missing data	*216 (100)*	*140 (100)*
**Relationship Status #**	Partner	0 (0)	0 (0)
	No partner	0 (0)	0 (0)
	Missing data	*216 (100)*	*140 (100)*
**Professional status * #**	Inactive	0 (0)	0 (0)
	Active	0 (0)	0 (0)
	Missing data	*216 (100)*	*140 (100)*
**Working in Healthcare #**	No	0 (0)	0 (0)
	Yes	0 (0)	0 (0)
	Missing data	*216 (100)*	*140 (100)*
**Educational Level #**	Low	0 (0)	0 (0)
	Medium	0 (0)	0 (0)
	High	0 (0)	0 (0)
	Missing data	*216 (100)*	*140 (100)*
**Smoking during pregnancy ** #**	Yes	0 (0)	0 (0)
	No	0 (0)	0 (0)
	Missing data	*216 (100)*	*140 (100)*
**History of positive SARS-CoV-2 test**	Yes	21 (9.7) ***	17 (12.1) ***
	No	129 (59.7)	84 (60.0)
	Missing data	*66 (30.6)*	*39 (27.9)*
**Chronic illness requiring medication #**	Yes	0 (0)	0 (0)
	No	0 (0)	0 (0)
	Missing data	*216 (100)*	*140 (100)*
**Gravidity**	Primigravida	80 (37.0)	NA
	Multigravida	71 (32.9)	NA
	Missing data	65 (30.1)	NA
**Gestational trimester**	1st trimester	41 (19.0) ***	NA
	2nd trimester	49 (22.7) ***	NA
	3rd trimester	61 (28.2) ***	NA
	Missing data	65 (30.1)	NA
**Breastfeeding**	Yes	NA	81 (57.9) ***
	No	NA	36 (25.7)
	Missing data	NA	*23 (16.4)*
**Participated in the COVID survey in 2020**	Yes	3 (1.4)	3 (2.1)
	No	152 (70.4)	126 (90.0)
	Do not know	5 (2.3)	11 (7.9)

	Missing data	56 (25.9)	0 (0)

Results are expressed as absolute numbers (%). NA = question was not applicable.

* At the time of survey completion or before maternity leave. ** Smoking during current/previous pregnancy for pregnant and post-partum, respectively.

# The question was asked after the question on vaccination status. When considering the order of the questions in both surveys, women who did not answer about their vaccination status had a missing response rate of 30% and they stopped the survey completely from the question “Has any member of your family or close relation refused COVID-19 vaccination?”, which stands two questions before the one on vaccination status. They therefore did not answer all the following questions, including those concerning their basic socio-professional characteristics, which did not allow for a more detailed comparative analysis of their characteristics.

*** These characteristics are different from the characteristics from the women included in the study by performing Chi^2 tests ($p<0.01$).

Supplementary Table S2: Association between socio-demographic, medical, obstetrical and ideological characteristics, and COVID-19 vaccination or willingness to get vaccinated among pregnant and postpartum women

	**Pregnant women with available vaccination status N=3194**				**Postpartum women with available vaccination status N=1659**			
**Determinants**	Not willing to get vaccinated N=2131 N (%)	Vaccinated or willing to get vaccinated N=1063 N (%)	**OR (95% CI)**	**aOR$_1$ (95% CI)**	Not willing to get vaccinated N=343 N (%)	Vaccinated or willing to get vaccinated N=1316 N (%)	**OR (95% CI)**	**aOR$_2$ (95% CI)**
**SOCIO-DEMOGRAPHIC CHARACTERISTICS**								
**Country**								
Belgium	58 (2.7)	240 (22.6)	**15.1 (11.3-20.7)**	**63.5 (32.3-131.8)**	28 (8.2)	168 (12.8)	1.20 (0.93-2.19)	**3.24 (1.22-9.81)**
Norway	1866 (87.6)	510 (48.0)	**Ref**	**Ref**	211 (61.5)	902 (68.5)	**Ref**	**Ref**
Switzerland	112 (5.3)	53 (5.0)	**1.73 (1.22-2.42)**	**4.01 (2.24-7.32)**	58 (16.9)	82 (6.2)	**0.33 (0.23-0.48)**	0.78 (0.35-1.82)
Netherlands	46 (2.2)	77 (7.2)	**6.12 (4.21-9.00)**	**27.6 (12.8-62.8)**	34 (9.9)	90 (6.8)	**0.62 (0.41-0.96)**	**7.51 (2.63-23.4)**
United Kingdom	49 (2.3)	183 (17.2)	**13.7 (9.90-19.2)**	**25.4 (13.3-51.0)**	12 (3.5)	74 (5.6)	1.44 (0.79-2.84)	1.37 (0.42-5.24)
**Maternal age**								
18-35	1804 (85.4)	871 (84.0)	Ref	⊥	277 (83.9)	1082 (84.1)	Ref	⊥
>35	308 (14.6)	166 (16.0)	1.12 (0.91-1.37)	⊥	53 (12.7)	204 (15.9)	0.99 (0.71-1.38)	⊥
**Working in healthcare**								
No	1298 (70.5)	581 (61.6)	Ref	Ref	200 (71.9)	747 (64.7)	Ref	Ref

Yes	544 (29.5)	362 (38.4)	**1.49 (1.26-1.75)**	1.24 (0.91-1.69)	78 (28.1)	408 (35.3)	**1.40 (1.05-1.88)**	1.21 (0.71-2.10)
**Employment at risk of contracting COVID-19**								
No	152 (53.1)	278 (54.6)	Ref	⊥	56 (50.5)	170 (44.2)	Ref	⊥
Yes	134 (46.9)	231 (45.4)	0.94 (0.70-1.26)	⊥	55 (49.5)	215 (55.8)	1.29 (0.84-1.96)	⊥
**Educational level**								
Low	63 (3.0)	10 (1.0)	Ref	Ref	10 (3.1)	24 (1.9)	Ref	⊥
Medium	406 (19.4)	134 (12.9)	**2.07 (1.08-4.41)**	1.15 (0.30-5.33)	91 (28.0)	199 (15.6)	0.91 (0.40-1.93)	⊥
High	1625 (77.6)	891 (86.1)	**3.45 (1.85-7.19)**	1.33 (0.36-5.89)	224 (68.9)	1055 (82.6)	1.96 (0.88-4.05)	⊥
**PERSONAL AND FAMILY HISTORY OF COVID-19**								
**History of personal positive SARS-CoV-2 test**								
No	2046 (96.0)	1005 (94.6)	Ref	⊥	317 (92.4)	1259 (95.7)	**Ref**	Ref
Yes	85 (4.0)	57 (5.4)	1.37 (0.96-1.92)	⊥	26 (7.6)	57 (4.3)	**0.55 (0.35-0.91)**	0.57 (0.22-1.67)
**History of personal COVID infection (suspected or confirmed)**								
No	1914 (89.8)	933 (87.9)	Ref	⊥	270 (82.8)	1114 (86.6)	Ref	⊥
Yes	217 (10.2)	129 (12.1)	1.22 (0.97-1.53)	⊥	56 (17.2)	173 (13.4)	0.75 (0.54-1.05)	⊥
**History of positive SARS-CoV-2 test in household or close family**								
No	1865 (87.5)	851 (80.1)	**Ref**	Ref	268 (78.1)	1072 (81.5)	Ref	⊥
Yes	266 (12.5)	212 (19.9)	**1.75 (1.43-2.13)**	0.95 (0.63-1.42)	75 (21.9)	244 (18.5)	0.81 (0.61-1.09)	⊥
**History of COVID-19 with hospitalization in ICU in household or close family among positive cases in family**								
No	111 (92.5)	53 (77.9)	**Ref**	*	58 (96.7)	82 (90.1)	Ref	⊥

Yes	9 (7.5)	15 (22.1)	**3.49 (1.46-8.80)**		2 (3.3)	9 (9.9)	2.91 (0.97-16.8)	⊥
MEDICAL AND OBSTETRICAL CHARACTERISTICS								
**Allergy requiring medication**								
No	909 (67.8)	507 (71.8)	Ref	⊥	164 (81.2)	626 (76.7)	Ref	⊥
Yes	431 (32.2)	199 (28.2)	0.83 (0.68-1.01)	⊥	38 (18.8)	190 (23.3)	1.31 (0.90-1.95)	⊥
**Chronic illness requiring medication**								
No	909 (66.7)	507 (62.2)	**Ref**	**Ref**	164 (78.1)	626 (70.3)	**Ref**	**Ref**
Yes	453 (33.3)	423 (37.8)	**1.22 (1.02-1.46)**	**1.47 (1.11-1.94)**	46 (21.9)	264 (29.7)	**1.50 (1.06-2.17)**	**2.16 (1.19-4.11)**
**Gravidity**								
Multigravida	1052 (49.4)	535 (50.3)	Ref	⊥	NA	NA		
Primigravida	1079 (50.6)	528 (49.7)	0.96 (0.83-1.11)	⊥				
**Trimester of pregnancy**								
1st trimester	185 (8.7)	166 (15.6)	Ref	Ref				
2nd trimester	674 (31.6)	428 (40.3)	**0.71 (0.56-0.90)**	0.75 (0.49-1.15)	NA	NA		
3rd trimester	1272 (59.7)	469 (44.1)	**0.41 (0.32-0.52)**	**0.30 (0.20-0.45)**				
**Breastfeeding**								
No	NA				16 (4.7)	120 (9.1)	**Ref**	**Ref**
Yes	NA				327 (95.3)	1196 (90.9)	**0.49 (0.28-0.81)**	**0.28 (0.09-0.81)**
BELIEFS ON COVID-19 AND VACCINES (AGAINST FLU AND COVID-19)								
**Belief that COVID-19 is more severe during pregnancy**					**Belief that COVID-19 is riskier during breastfeeding**			

No	638 (29.9)	142 (13.4)	**Ref**	**Ref**	268 (78.1)	1072 (81.5)	Ref	⊥
Yes	1493 (70.1)	921 (86.6)	**2.77 (2.28-3.39)**	**1.65 (1.20-2.29)**	75 (21.9)	244 (18.5)	0.81 (0.61-1.09)	⊥
**Had flu vaccine last winter**								
No	1511 (70.9)	557 (52.4)	**Ref**	**Ref**	205 (59.8)	394 (29.9)	Ref	Ref
Yes	620 (29.1)	506 (47.6)	**2.21 (1.90-2.58)**	**1.43 (1.05-1.95)**	138 (40.2)	922 (70.1)	**3.48 (2.72-4.45)**	**3.25 (1.93-5.52)**
**Effectiveness of COVID vaccine for mother and unborn child**					**Effectiveness of COVID vaccine to protect mother and breastfed child**			
Not very + not at all effective	458 (21.5)	17 (1.6)	**Ref**	**Ref**	128 (37.3)	31 (2.4)	**Ref**	Ref
Extremely + very effective + effective	1673 (78.5)	1046 (98.4)	**16.9 (10.7-28.6)**	**2.39 (1.15-5.27)**	215 (62.7)	1285 (97.6)	**24.7 (16.4-38.1)**	2.49 (0.84-7.64)
**Safety of COVID vaccine for mother and unborn child**					**Safety of COVID vaccine for mother and child while breastfeeding**			
Not very + not at all safe	1125 (52.8)	62 (5.8)	**Ref**	**Ref**	219 (63.8)	38 (2.9)	**Ref**	**Ref**
Extremely + very + safe	1006 (47.2)	1001 (94.2)	**18.1 (13.9-23.9)**	**36.5 (21.9-63.8)**	124 (36.2)	1278 (97.1)	**62.7 (41.9-96.7)**	**72.4 (35.4-161.2)**
TRUST IN THE FOLLOWING SOURCES TO PROVIDE RELIABLE INFORMATION REGARDING THE CORONAVIRUS								
**Great or absolute trust in public authorities (government and health authorities)**								
No	754 (35.7)	465 (44.8)	**Ref**	Ref	200 (60.6)	464 (36.0)	**Ref**	Ref
Yes	1359 (64.3)	574 (55.2)	**0.68 (0.59-0.80)**	0.75 (0.54-1.05)	130 (39.4)	824 (64.0)	**2.73 (2.13-3.51)**	1.12 (0.61-1.99)
**Great or absolute trust in healthcare providers (general practitioner, obstetrician, midwife, and pharmacist)**								
No	1124 (53.2)	528 (50.8)	Ref	⊥	220 (66.7)	636 (49.4)	**Ref**	Ref
Yes	989 (46.8)	511 (49.2)	1.10 (0.95-1.28)	⊥	110 (33.3)	652 (50.6)	**2.05 (1.59-2.65)**	0.69 (0.40-1.15)
**Great or absolute trust in non-professional sources of information**								

No	2071 (98.0)	1018 (98.0)	Ref	⊥	324 (98.2)	1259 (97.7)	Ref	⊥
Yes	42 (2.0)	21 (2.0)	1.02 (0.59-1.71)	⊥	6 (1.8)	29 (2.3)	1.24 (0.55-3.34)	⊥
**History of a family member who refused COVID-19 vaccination**								
No	1693 (79.4)	845 (79.5)	Ref	⊥	210 (61.2)	1049 (79.7)	**Ref**	Ref
Yes	438 (20.6)	218 (20.5)	1.00 (0.83-1.20)	⊥	133 (38.8)	267 (20.3)	**0.40 (0.31-0.52)**	0.68 (0.40-1.19)
**Belief that restrictive measures are very / extremely useful to prevent the pandemic (disinfectant, mask, social distance, working from home, curfew, lockdown)**								
No	167 (7.8)	175 (16.5)	**Ref**	Ref	79 (23.0)	149 (11.3)	**Ref**	Ref
Yes	1964 (92.2)	21 (83.5)	**0.43 (0.34-0.54)**	1.09 (0.60-1.95)	264 (77.0)	1167 (88.7)	**2.24 (1.72-3.17)**	0.94 (0.39-2.24)
**Belief that vaccination is very / extremely useful to prevent the pandemic**								
No	652 (30.6)	220 (20.7)	**Ref**	**Ref**	219 (63.8)	245 (18.6)	**Ref**	Ref
Yes	1479 (69.4)	843 (79.3)	**1.69 (1.42-2.01)**	**1.79 (1.22-2.66)**	124 (36.2)	1071 (81.4)	**7.72 (5.96-10.0)**	1.82 (0.95-3.41)

ICU: Intensive Care Unit; NA = question was not applicable; OR = crude odds ratio

The bold numbers indicate ORs not including 1.

⊥ As these variables were not significantly associated with vaccine willingness in the univariate analyses, they were not included in the multivariable models.

aOR_1 = adjusted odds ratio, adjusted for country, working in healthcare, highest education level, positive COVID test in the family, chronic illness, trimester of pregnancy, history of flu vaccine the previous winter, belief that the COVID vaccine is effective, belief that the COVID vaccine is safe during pregnancy, trust in public authorities, belief that restrictive measures are useful, and belief that vaccination is useful

aOR_2 = adjusted for country, working in healthcare, history of positive SARS-CoV-2 test, chronic illness, breastfeeding, history of flu vaccine the previous winter, belief that the COVID vaccine is effective, belief that the COVID vaccine is safe during breastfeeding, trust in public authorities, trust in healthcare providers, belief that restrictive measures are useful, and belief that vaccination is useful.

* The variable "History of COVID-19 with hospitalization in ICU in household or close family" was not added in model 1 because of collinearity with the variable "History of positive COVID test in household or close family". Among both, the variable "History of positive COVID test in household or close family" was not chosen to be included in model 1 because there were 3044 observations deleted because of missingness with the variable "History of COVID-19 with hospitalization in ICU in household or close family".

Supplementary Table 3: Association between socio-demographic, medical, obstetrical and ideological characteristics, and COVID-19 vaccine willingness, whatever the vaccination status, among pregnant women

**Pregnant women N=2710**<br><br>**Determinants**	**Not willing to get vaccinated N=1552**<br><br>**N (%)**	**Do not know N=658**<br><br>**N (%)**	**Willing to get vaccinated N=500**<br><br>**N (%)**	**p-value**
SOCIO-DEMOGRAPHIC CHARACTERISTICS				
**Country**				
Belgium	42 (2.7)	16 (2.4)	24 (4.8)	0.04
Norway	1357 (87.4)	588 (89.4)	431 (86.2)	Ref
Switzerland	82 (5.3)	30 (4.6)	16 (3.2)	0.20
The Netherlands	36 (2.3)	18 (1.5)	18 (3.6)	0.30
United Kingdom	35 (2.3)	11 (2.1)	11 (2.2)	0.64
**Maternal age**				
18-35	1324 (86.0)	553 (84.8)	424 (85.3)	
>35	215 (14.0)	99 (15.2)	73 (14.7)	0.74
**Relationship status**				
Partner	1507 (97.9)	640 (98.2)	489 (98.4)	
No partner	32 (2.1)	12 (1.8)	8 (1.6)	0.79
**Professional status**				
Inactive	204 (13.3)	61 (9.4)	56 (11.3)	
Active	1333 (86.7)	591 (90.6)	441 (88.7)	0.03
**Working in healthcare**				
No	879 (66.4)	432 (73.5)	297 (67.7)	
Yes	444 (33.6)	156 (26.5)	142 (32.3)	0.009
**Educational level**				
Low	47 (3.1)	16 (2.5)	7 (1.4)	Ref
Medium	321 (21.0)	96 (14.8)	73 (14.7)	0.50
High	1158 (75.9)	535 (82.7)	417 (83.9)	0.07
PERSONAL AND FAMILY HISTORY OF COVID-19				
**History of personal positive SARS-CoV-2 test**				
No	1486 (95.7)	635 (96.5)	484 (96.8)	
Yes	66 (4.3)	23 (3.5)	16 (3.2)	0.49
**History of personal COVID infection (suspected or confirmed)**				

No	1390 (89.6)	597 (90.7)	462 (92.4)	
Yes	162 (10.4)	61 (9.3)	38 (7.6)	0.16
**History of positive SARS-CoV-2 test in household or close family**				
No	1355 (87.3)	581 (88.3)	440 (88.0)	
Yes	197 (12.7)	77 (11.7)	60 (12.0)	0.79
MEDICAL AND OBSTETRICAL CHARACTERISTICS				
**Smoking during pregnancy**				
No	1517 (98.6)	648 (99.4)	487 (98.0)	
Yes	22 (1.4)	20 (0.6)	10 (2.0)	0.11
**Chronic illness requiring medication**				
No	665 (55.3)	278 (53.1)	202 (47.1)	
Yes	537 (44.7)	246 (46.9)	227 (52.9)	0.01
**Gravidity**				
Multigravida	760 (49.0)	323 (49.1)	350 (46.8)	
Primigravida	792 (51.0)	335 (50.9)	286 (53.2)	0.67
**Trimester of pregnancy**				
1st trimester	105 (6.8)	116 (17.6)	67 (13.4)	Ref
2nd trimester	488 (31.4)	226 (34.3)	207 (41.4)	10^{-7}
3rd trimester	959 (61.8)	316 (48.0)	226 (45.2)	10^{-17}
BELIEFS ON VACCINES (AGAINST FLU AND COVID-19)				
**Had flu vaccine last winter**				
No	1082 (69.7)	458 (69.6)	297 (59.4)	
Yes	470 (30.3)	200 (30.4)	203 (40.6)	5.10^{-5}
**Effectiveness of COVID vaccine for mother and unborn child**				
Not very + not at all effective	420 (27.1)	40 (6.1)	5 (1.0)	
Extremely + very effective + effective	1132 (72.9)	618 (93.9)	495 (99.0)	$<10^{-16}$
**Safety of COVID vaccine for mother and unborn child**				
Not very + not at all safe	961 (61.9)	180 (27.4)	15 (3.0)	

Extremely + very + safe	591 (38.1)	478 (72.6)	485 (97.0)	$<10^{-16}$
**TRUST IN THE FOLLOWING SOURCES TO PROVIDE RELIABLE INFORMATION REGARDING THE CORONAVIRUS**				
**Great or absolute trust in public authorities (government and health authorities)**				
No	581 (37.8)	195 (29.9)	130 (26.1)	
Yes	958 (62.2)	458 (70.1)	368 (73.9)	7.10^{-7}
**Great or absolute trust in healthcare providers (general practitioner, obstetrician, midwife, and pharmacist)**				
No	835 (54.3)	326 (49.9)	251 (50.4)	
Yes	704 (45.7)	327 (50.1)	247 (49.6)	0.10
**Great or absolute trust in non-professional sources of information**				
No	1505 (97.8)	642 (98.3)	485 (97.4)	
Yes	34 (2.2)	11 (1.7)	13 (2.6)	0.55
**History of a family member who refused COVID-19 vaccination**				
No	1210 (78.0)	546 (83.0)	412 (82.4)	
Yes	342 (22.0)	112 (17.0)	88 (17.6)	0.009
**Belief that restrictive measures are very / extremely useful to prevent the pandemic (disinfectant, mask, social distance, working from home, curfew, lockdown)**				
No	137 (8.8)	31 (4.7)	28 (5.6)	
Yes	1415 (91.2)	627 (95.3)	472 (94.4)	0.0009
**Belief that vaccination is very / extremely useful to prevent the pandemic**				
No	514 (33.1)	153 (23.3)	61 (12.2)	
Yes	1038 (66.9)	505 (76.7)	439 (87.8)	$<10^{-16}$

This table displays the first sensitivity analysis with three levels in the outcome variable.
ICU: Intensive Care Unit; NA = question was not applicable; OR = crude odds ratio
The percentages are displayed in columns. Chi^2 tests were performed by row to assess whether there was an association between socio-demographic, medical and obstetrical characteristics of the participants and the three levels of response regarding their willingness to be vaccinated against COVID-19: “not willing to get vaccinated”, “I don’t know” and “willing to get vaccinated”.

Supplementary Table S4: Association between socio-demographic, history of COVID-19, medical and obstetrical characteristics, and COVID-19 vaccination or willingness to get vaccinated restricted to breastfeeding women in the postpartum women group

	**Breastfeeding women with available vaccination status N=1523**			
**Determinants**	Not willing to get vaccinated N (%)	Vaccinated or willing to get vaccinated N (%)	**OR (95% CI)**	**aOR (95% CI)**
**Country**				
Belgium	23 (7.0)	139 (11.6)	1.46 (0.93-2.38)	**3.07 (1.10-9.98)**
Norway	207 (63.3)	858 (71.7)	Ref	**Ref**
Switzerland	55 (16.8)	61 (5.1)	**0.27 (0.18-0.40)**	0.78 (0.33-1.97)
Netherlands	33 (10.1)	83 (6.9)	**0.61 (0.40-0.94)**	**6.11 (2.04-20.1)**
United Kingdom	9 (2.8)	55 (4.6)	1.47 (0.75-3.24)	2.15 (0.53-10.36)
**Maternal age**				
18-35	265 (83.9)	986 (83.8)	Ref	⊥
>35	51 (16.1)	190 (16.2)	1.00 (0.72-1.41)	⊥
**Professional status**				
Inactive	267 (84.8)	1057 (90.4)	Ref	Ref
Active	48 (15.2)	112 (9.6)	**1.70 (1.17-2.43)**	***
**Working in healthcare**				
No	191 (71.8)	690 (65.0)	Ref	Ref
Yes	75 (28.2)	371 (35.0)	**1.37 (1.02-1.85)**	1.07 (0.62-1.88)
**Employment at risk of contracting COVID-19**				
No	54 (52.4)	141 (43.5)	Ref	⊥
Yes	49 (47.6)	183 (56.5)	1.43 (0.92-2.24)	⊥
**Educational level**				
Low	10 (3.2)	20 (1.7)	**Ref**	Ref
Medium	87 (27.9)	164 (14.0)	0.94 (0.41-2.06)	4.19 (0.43-46.6)
High	215 (68.9)	986 (84.3)	**2.29 (1.02-4.86)**	7.00 (0.76-74.4)
**PERSONAL AND FAMILY HISTORY OF COVID-19**				
**History of personal positive SARS-CoV-2 test**				
No	303 (92.7)	1145 (95.7)	Ref	Ref
Yes	24 (7.3)	51 (4.3)	**0.56 (0.34-0.94)**	0.56 (0.20-1.77)
**History of personal COVID infection (suspected or confirmed)**				
No	258 (82.7)	1023 (86.8)	Ref	⊥
Yes	54 (17.3)	155 (13.2)	0.72 (0.52-1.02)	⊥
**History of personal COVID infection with hospitalization in ICU**				
No	54 (98.2)	61 (100)	Ref	⊥
Yes	1 (1.8)	0 (0)	*	⊥
**History of positive SARS-CoV-2 test in household or close family**				
No	260 (79.5)	982 (82.1)	Ref	⊥

Yes	67 (20.5)	214 (17.9)	0.85 (0.63-1.55)	⊥
**History of COVID-19 with hospitalization in ICU in household or close family**				
No	55 (96.5)	61 (87.1)	Ref	⊥
Yes	2 (3.5)	9 (12.9)	4.06 (0.99-27.4)	⊥
MEDICAL CHARACTERISTICS				
**Allergy**				
No	158 (82.3)	565 (76.4)	Ref	⊥
Yes	34 (17.7)	175 (23.6)	1.44 (0.97-2.19)	⊥
**Chronic illness requiring medication**				
No	158 (80.2)	565 (70.8)	**Ref**	**Ref**
Yes	39 (19.8)	233 (29.2)	**1.67 (1.15-2.48)**	**2.14 (1.15-4.18)**
BELIEFS ON VACCINES (AGAINST FLU AND COVID-19)				
**Belief that COVID is risky during breastfeeding**				
No	260 (79.5)	982 (82.1)	Ref	⊥
Yes	67 (20.5)	214 (17.9)	0.85 (0.62-1.15)	⊥
**Had flu vaccine last winter**				
No	194 (59.3)	365 (30.5)	Ref	Ref
Yes	133 (40.7)	831 (69.5)	**3.32 (2.58-4.28)**	**3.19 (1.86-5.52)**
**Effectiveness of COVID vaccine to protect mother and breastfed child**				
Not very + not at all effective	118 (36.1)	28 (2.3)	**Ref**	Ref
Extremely + very + effective	209 (63.9)	1168 (97.7)	**23.6 (15.4-37.1)**	2.16 (0.69-7.09)
**Safety of COVID vaccine for mother and child while breastfeeding**				
Not very + not at all safe	207 (63.3)	32 (2.7)	Ref	Ref
Extremely + very + safe	120 (36.7)	1164 (97.3)	**62.7 (41.9-96.7)**	**82.0 (38.1-194)**
TRUST IN THE FOLLOWING SOURCES TO PROVIDE RELIABLE INFORMATION REGARDING THE CORONAVIRUS				
**Great or absolute trust in public authorities (government and health authorities)**				
No	187 (59.2)	399 (33.9)	**Ref**	**Ref**
Yes	129 (40.8)	779 (66.1)	**2.83 (2.19-3.66)**	1.02 (0.54-1.86)
**Great or absolute trust in healthcare providers (GP, obstetrician, midwife, pharmacist)**				
No	209 (66.1)	577 (49.0)	**Ref**	Ref
Yes	107 (33.9)	601 (51.0)	**2.03 (1.57-2.64)**	0.63 (0.36-1.07)
**Great or absolute trust in non-professional sources of information**				
No	310 (98.1)	1152 (97.8)	Ref	⊥
Yes	6 (1.9)	26 (2.2)	1.17 (0.51-3.15)	⊥
**Family member refused COVID vaccine**				
No	206 (63.0)	956 (79.9)	**Ref**	Ref
Yes	121 (37.0)	240 (20.1)	**0.43 (0.33-0.56)**	0.75 (0.43-1.35)
**Family member decision to refuse a COVID vaccine contributed to the perception of vaccination**				
No	106 (87.6)	221 (92.1)	Ref	⊥

Yes	15 (12.4)	19 (7.9)	0.61 (0.30-1.26)	⊥
**Belief that restrictive measures are very / extremely useful to prevent the pandemic (disinfectant, mask, social distance, working from home, curfew, lockdown)**				
No	70 (21.4)	119 (9.9)	**Ref**	Ref
Yes	257 (78.6)	1077 (90.1)	**2.47 (1.78-3.40)**	0.69 (0.26-1.80)
**Belief that vaccination is very / extremely useful to prevent the pandemic**				
No	205 (62.7)	204 (17.1)	**Ref**	Ref
Yes	122 (37.3)	992 (82.9)	**8.17 (6.25-10.73)**	1.78 (0.90-3.44)

This table displays the second sensitivity analysis.
ICU: Intensive Care Unit; NA = question was not applicable; OR = crude odds ratio
The bold numbers indicate ORs not including 1.
aOR = adjusted odds ratio, adjusted for country, maternal age, professional status, working in healthcare, education level, personal history of positive COVID test, history of flu vaccine the previous winter, belief that the COVID vaccine is effective, belief that the COVID vaccine is safe during pregnancy, great or absolute trust in public authorities (government and health authorities), great or absolute trust in healthcare providers (GP, obstetrician, midwife, pharmacist family member who refused the COVID vaccine, belief that restrictive measures are very / extremely useful to prevent the pandemic, belief that vaccination is very / extremely useful to prevent the pandemic.
* The model did not run because of the absence of event.
⊥ As these variables were not significantly associated with vaccine willingness in the univariate analyses, they were not included in the multivariable models.

Supplementary Table S5: Association between beliefs on the impact of COVID-19 infection and COVID-19 vaccine willingness among pregnant and postpartum women.

**Pregnant women**	**Not willing to get vaccinated N=2131**		**Vaccinated or willing to get vaccinated N=1063**	
	n/N	**% (95% CI)**	n/N	**% (95% CI)**
**Belief that coronavirus infection can be more severe during pregnancy than among non-pregnant women of the same age**	1493/2131	70.1 (68.1-72.0)	921/1063	86.6 (84.4-88.6)
**Specification in case of positive response ***				
Higher risk of maternal complication	1196/1250	95.7 (94.4-96.7)	787/794	99.1 (98.1-99.6)
Higher risk of congenital malformation	150/239	62.8 (56.3-68.9)	109/138	79.0 (71.2-85.5)
Impact on fetal growth and development	373/463	80.4 76.5-83.9)	257/296	86.8 (82.4-90.5)
Higher risk of pregnancy complication	854/904	94.5 (96.7-98.9)	688/702	98.0 (96.7-98.9)
Mother-to-child transmission during delivery	392/488	80.3 (76.5-83.7)	273/313	87.2 (83.0-90.7)
**Postpartum women**	**Not willing to get vaccinated N=343**		**Vaccinated or willing to get vaccinated N=1316**	
	**n/N**	**% (95% CI)**	**n/N**	**% (95% CI)**
**Belief that breastfeeding may be risky to extremely risky to the infant if the breastfeeding mother is infected with coronavirus**	217/343	63.3 (57.9-68.4)	818/1316	62.2 (59.5-64.8)
**Specification in case of positive response ***				
Disease could stop the mother from breastfeeding (feeling too sick)	58/105	55.2 (45.2-65.0)	120/189	63.5 (56.2-70.4)
Disease could negatively affect the milk production	48/97	49.5 (39.2-59.8)	96/172	55.8 (48.1-63.4)
Transmission of the virus to the baby through milk	41/89	46.1 (35.4-57.0)	73/147	49.7 (41.3-58.0)
Transmission of the virus to the baby while breastfeeding through close contact	84/128	65.6 (56.7-73.8)	221/285	77.5 (72.2-82.3)

CI: confidence interval

* Multiple answers possible

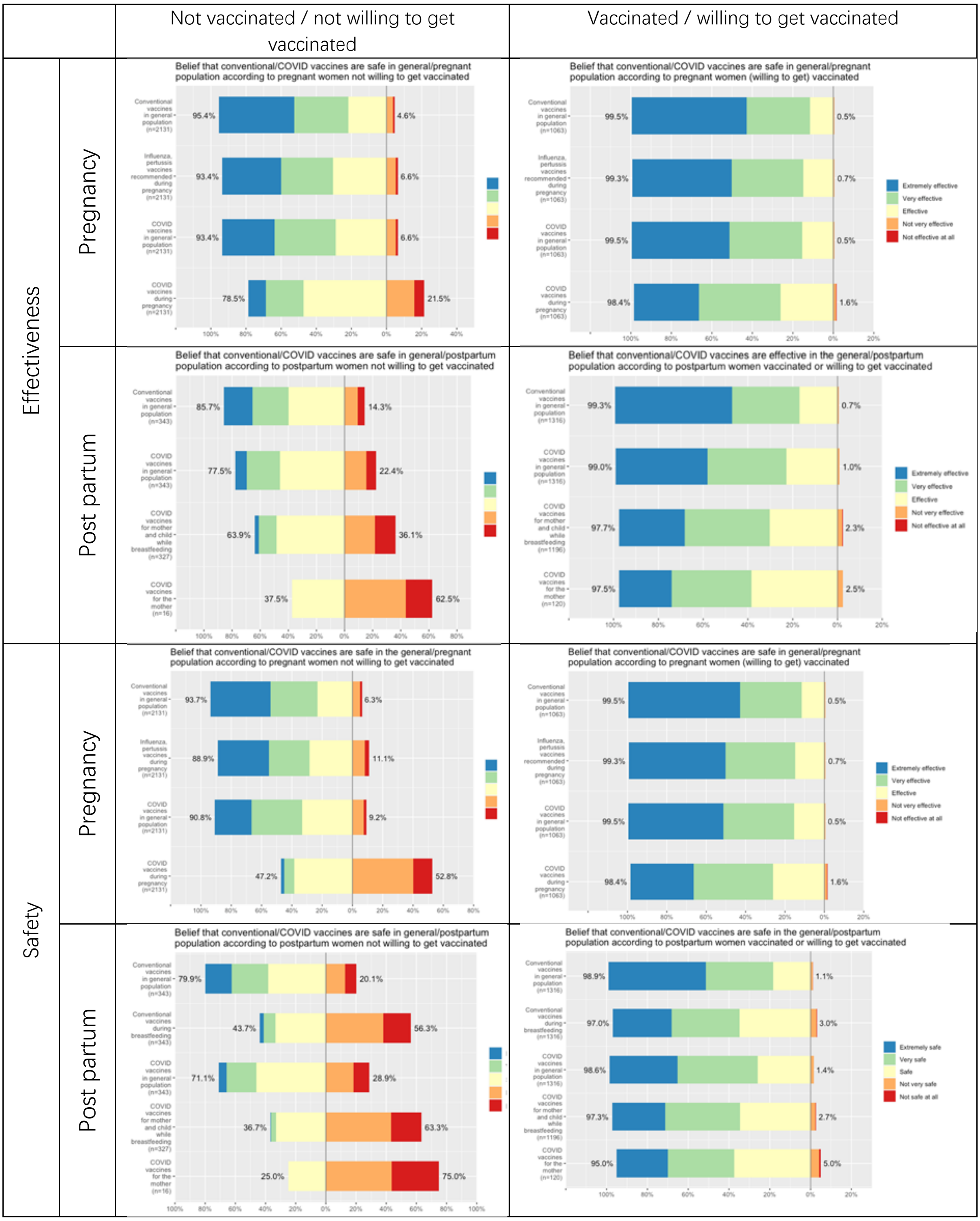

Supplementary Figure S1: Perception on the safety and effectiveness of conventional and COVID-19 vaccines among pregnant and postpartum women, vaccinated or willing to get vaccinated or not.

For postpartum women, the 2 last questions were mutually exclusive: breastfeeding mothers were asked for their perception on themselves and their child while breastfeeding; mothers feeding their child with formula were asked for their perception on themselves only (these 2 questions are merged in supplementary table S2).

	Pregnancy	Postpartum
Belgium	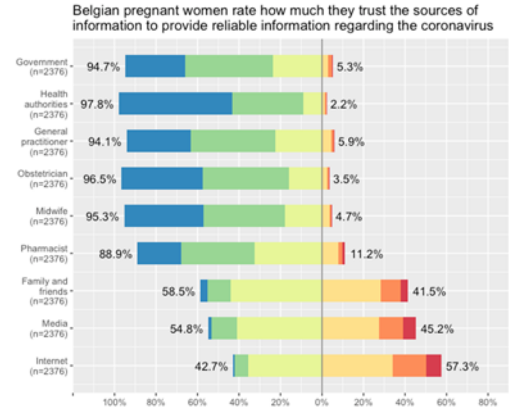	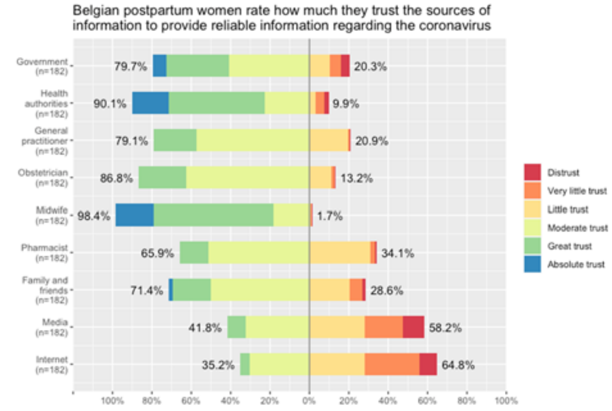
Norway	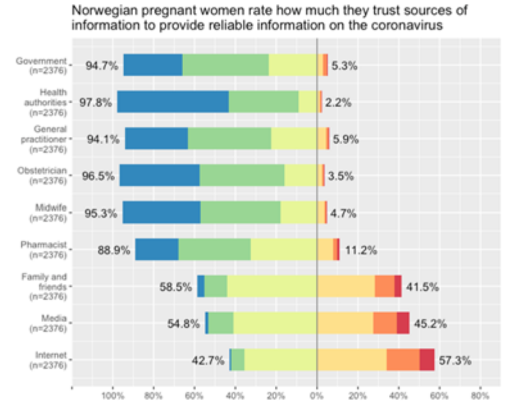	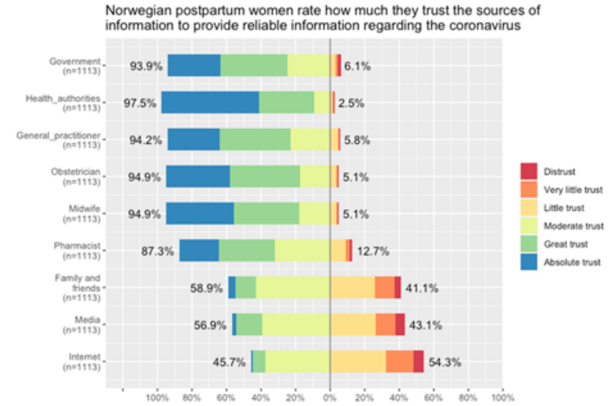
Switzerland	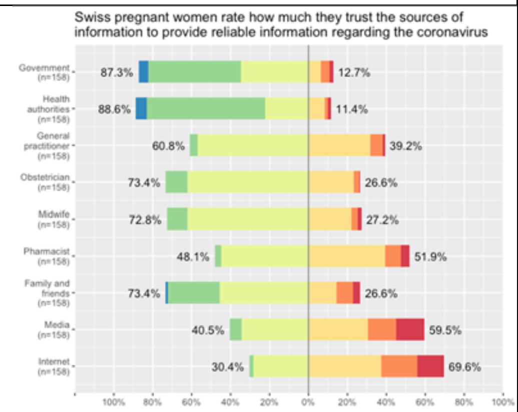	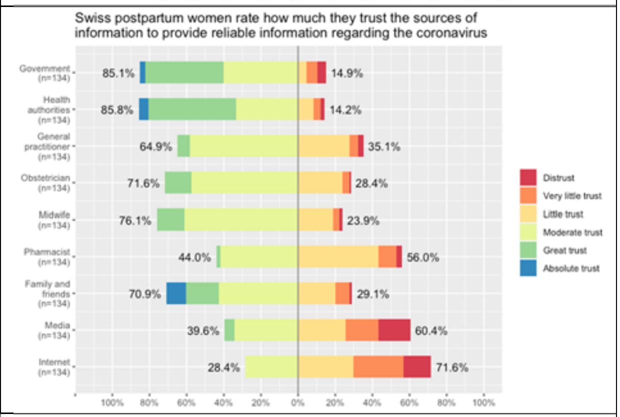
The Netherlands	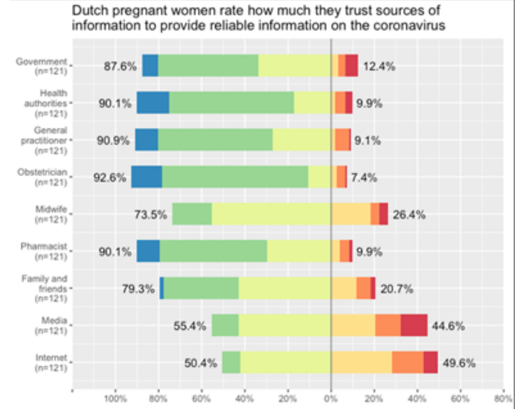	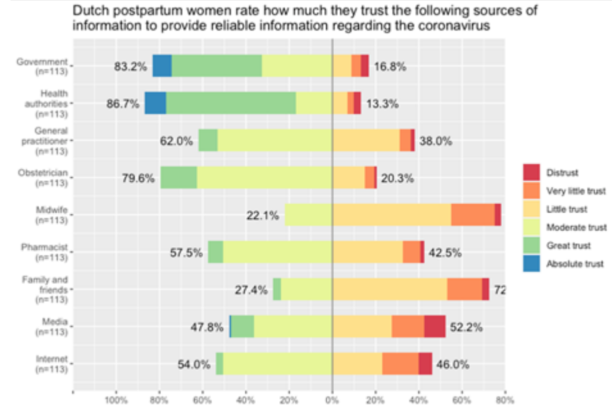

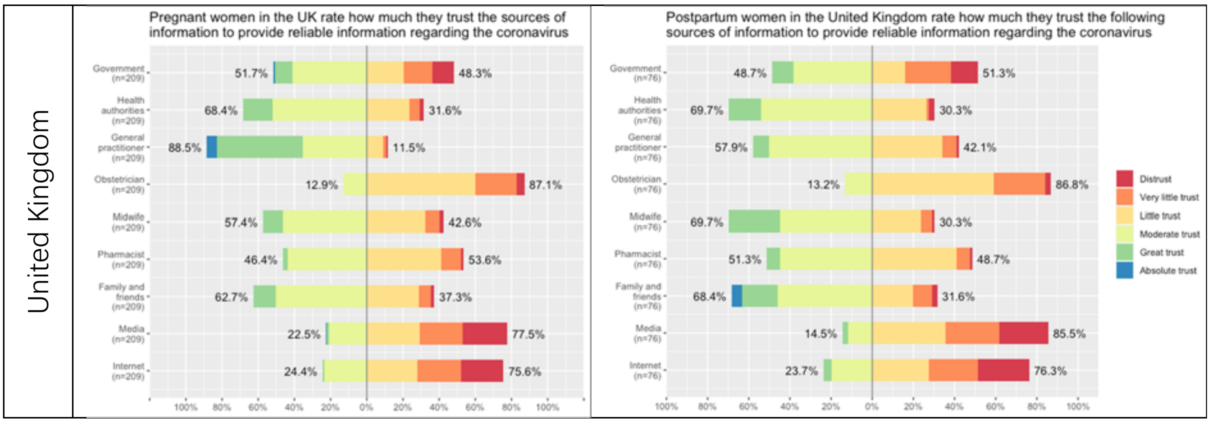

Supplementary Figure S2: Sources trusted to provide information regarding the coronavirus by obstetrical status and by country

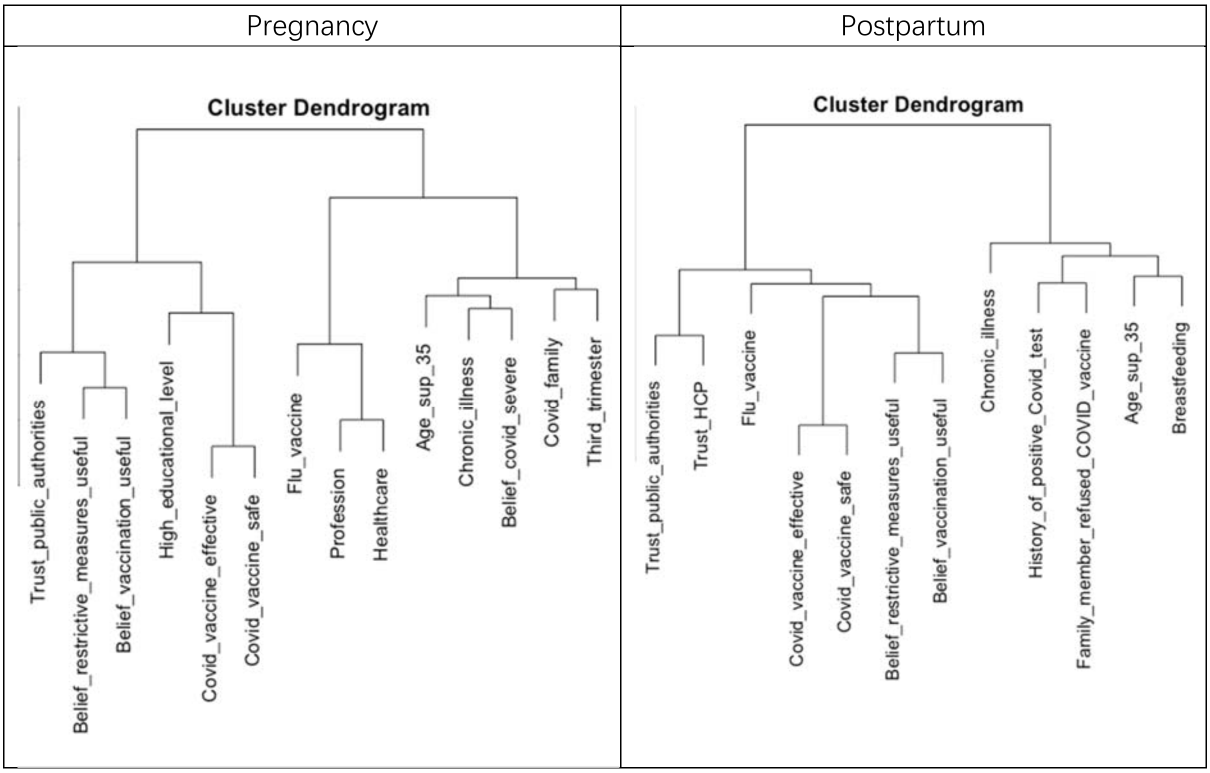

Supplementary Figure S3: Hierarchical cluster analysis of determinants associated with vaccination adhesion among pregnant (S3a) and postpartum women (S3b)

Hierarchical clustering is an algorithm that attempts to group subjects with similar features into clusters. These two dendrograms show the hierarchical relationship between determinants of vaccination willingness among pregnant and postpartum women. Traditionally, these factors are considered as confounding factors and can be addressed by multivariable regression modeling (Supplementary table 2). (Zhang Z, Murtagh F, Van Poucke S, Lin S, Lan P. Hierarchical cluster analysis in clinical research with heterogeneous study population: highlighting its visualization with R. Ann Transl Med 2017;5(4):75. doi: 10.21037/ atm.2017.02.05)

HCP: Health Care Providers
